# General transcription factor TAF4 antagonizes epigenetic silencing by Polycomb to maintain intestine stem cell functions

**DOI:** 10.1038/s41418-022-01109-6

**Published:** 2023-01-13

**Authors:** Susanna Säisä-Borreill, Guillaume Davidson, Thomas Kleiber, Andréa Thevenot, Elisabeth Martin, Stanislas Mondot, Hervé Blottière, Alexandra Helleux, Gabrielle Mengus, Michelina Plateroti, Isabelle Duluc, Irwin Davidson, Jean-Noel Freund

**Affiliations:** 1 University of Strasbourg, Inserm, UMR-S1113/IRFAC, FHU ARRIMAGE, FMTS, 67200 Strasbourg, France; 2 Institut de Génétique et de Biologie Moléculaire et Cellulaire, Department of Functional Genomics and Cancer, CNRS/Inserm/University of Strasbourg, 1 Rue Laurent Fries, 67404 Illkirch Cédex, France; 3 Orphazyme, Ole Maaloes 3, 2200 Copenhagen, Denmark; 4 University Paris-Saclay, INRAE, AgroParisTech, Micalis Institute, 78350 Jouy-en-Josas, France

**Keywords:** Molecular biology, Physiology

## Abstract

Taf4 (TATA-box binding protein-associated factor 4) is a subunit of the general transcription factor TFIID, a component of the RNA polymerase II pre-initiation complex that interacts with tissue-specific transcription factors to regulate gene expression. Properly regulated gene expression is particularly important in the intestinal epithelium that is constantly renewed from stem cells. Tissue-specific inactivation of Taf4 in murine intestinal epithelium during embryogenesis compromised gut morphogenesis and the emergence of adult-type stem cells. In adults, Taf4 loss impacted the stem cell compartment and associated Paneth cells in the stem cell niche, epithelial turnover and differentiation of mature cells, thus exacerbating the response to inflammatory challenge. *Taf4* inactivation ex vivo in enteroids prevented budding formation and maintenance and caused broad chromatin remodeling and a strong reduction in the numbers of stem and progenitor cells with a concomitant increase in an undifferentiated cell population that displayed high activity of the Ezh2 and Suz12 components of Polycomb Repressive Complex 2 (PRC2). Treatment of Taf4-mutant enteroids with a specific Ezh2 inhibitor restored buddings, cell proliferation and the stem/progenitor compartment. *Taf4* loss also led to increased PRC2 activity in cells of adult crypts associated with modification of the immune/inflammatory microenvironment that potentiated *Apc*-driven tumorigenesis. Our results reveal a novel function of Taf4 in antagonizing PRC2-mediated repression of the stem cell gene expression program to assure normal development, homeostasis, and immune-microenvironment of the intestinal epithelium.

## Introduction

The intestinal epithelium is characterized by dynamic cell renewal every 5–6 days fueled by active stem cells (SCs) located in crypts, the Lgr5^high^ Crypt Base Columnar cells (CBCs), that generate committed progenitors and ultimately mature digestive cells of the absorptive and secretory lineages [1]. These complex processes are controlled by complementary signaling pathways of which the main ones are Wnt, Notch and BMP. The last decades identified a whole range of transcription factors among which bHLH, HMG, homeodomain, Zinc-finger, Krüppel-like and other factors that are targeted by and/or cooperate with these pathways to ensure the dynamic homeostasis of the gut epithelium. These factors bind specific *cis*-regulatory DNA sequences and interact with the pre-initiation complex (PIC) of the general transcription machinery to promote gene expression. Their precise contribution to gut development and homeostasis has been deciphered through genetic knockouts. However, beyond these tissue-specifying transcription factors, the role of the general transcription machinery has not been addressed so far in the gut.

Taf4a (hereafter Taf4, for TATA-box binding protein-associated factor 4) and Taf4b are paralogous subunits of the general transcription factor TFIID comprising the TATA-box binding protein (TBP) and 13–14 TBP-associated factors (TAFs), that plays a critical role in PIC formation. While Taf4 is widely expressed, Taf4b displays a cell-restricted expression. We previously inactivated the *Taf4* gene in a variety of somatic murine tissues or during embryogenesis showing how Taf4 controlled gene expression programs in a tissue-specific manner regulating embryonic tissue differentiation [2] as well as the homeostasis of the epidermis [3] and the function of several endoderm-derived cell types such as the activation of post-natal metabolism genes in neonatal hepatocytes and the identity and activity of pancreatic beta cells [4, 5]. In addition to the liver and pancreas, the intestinal epithelium is another endodermal derivative that deserves attention because of its constant and active turnover.

Here, we inactivated *Taf4* in the intestinal epithelium in vivo during gut morphogenesis, adult homeostasis, and ex vivo in enteroid models. *Taf4* knockout compromised gut morphogenesis in embryos and impaired SCs and the dynamic homeostasis of the adult intestine. We reveal a novel function of Taf4 in antagonizing Polycomb Repressive Complex 2 (PRC2) to maintain the SC gene expression program. In absence of Taf4, SCs show increased PRC2 activity that correlates with an altered mucosal immune-microenvironment possibly involved in increased *Apc*-driven tumorigenesis.

## Results

### *Taf4* is required for proper development and morphogenesis of the embryonic intestinal endoderm

**Fig. 1:**
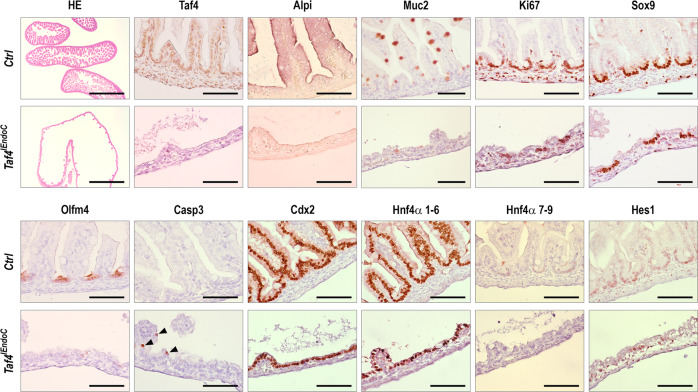
Morphological alteration resulting from *Taf4* inactivation in the gut endoderm of E18.5 fetuses. Morphology (HE) and immunohistochemical detection of the indicated proteins in E18.5 control *Taf4*^*los/lox*^ (Ctrl) and *Taf4*^*IEndoC*^ littermates. Bars are 50 µm except for HE and Muc2 where they represent 500 µm and 100 µm, respectively.

*Taf4* gene inactivation starting around days 10–11 post coitum (dpc) in the presumptive intestinal endoderm of *Taf4*^*lox/lox*^*::VilCre* embryos (hereafter *Taf4*^*IEndoC*^) led to perinatal lethality. Loss of Taf4 protein was progressive, being mosaic at 14.5 dpc and almost complete at 17.5 dpc. At this stage, the length of the gut was reduced in mutant compared to control fetuses (3.29 vs. 4.52 cm, *p* = 0.0007) with impaired morphogenesis and altered alkaline phosphatase activity and Muc2 expression indicated perturbed cell differentiation, whereas cell proliferation labeled by Ki67 was unchanged. The impact of *Taf4* loss on the dynamic morphogenetic process was enhanced at E18.5 as the mucosa became flat with only few bulged villi (Fig. 1). Alkaline phosphatase (Alpi) activity and mucin Muc2 expression were strongly reduced. Nevertheless, presumptive crypt regions were preserved as evidenced by labeling with Sox9 and Ki67 that further demonstrated the proliferative capacity of these cells. However, Olfm4 marking the emergence of adult-type SCs in the inter-villi regions was barely detectable. By contrast, expression of Cdx2 and Hnf4α 1–6, two important transcription factors for intestinal identity and differentiation, was unaltered, whereas Hes1 and Hnf4α 7–9 were diminished and irregular in the regions presenting the most altered phenotype. In addition, Caspase-3-stained apoptotic cells were observed mainly at the level of remnant villi.

### *Taf4* regulates the dynamic homeostasis of the adult intestinal epithelium

**Fig. 2:**
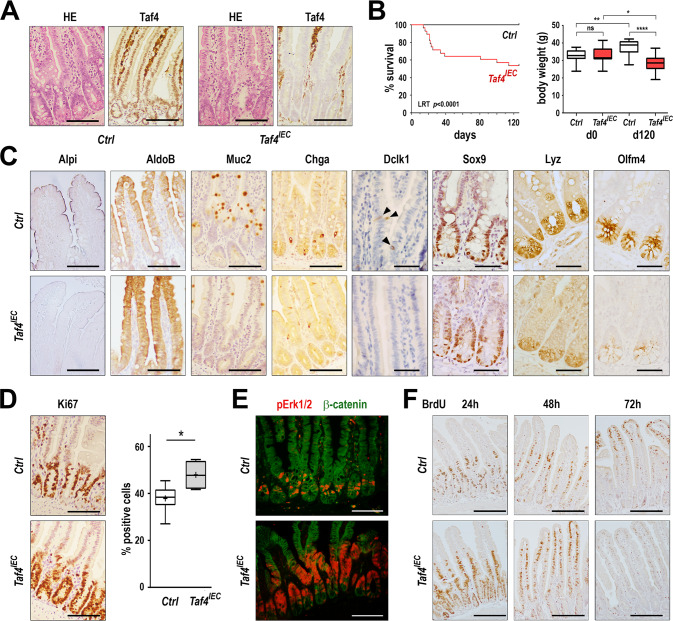
Homeostasis defects induced by *Taf4* inactivation in the adult gut epithelium. **A** Histology (HE) and immunodetection of the Taf4 protein in the intestine of adult *Taf4*^*IEC*^ and control *Taf4*^*los/lox*^ (Ctrl) mice 10 days after Tamoxifen injection. Bars are 100 µm. **B** Overall survival of *Taf4*^*IEC*^ (red boxes) and *Taf4*^*lox/lox*^ (Ctrl; white boxes) mice after Tamoxifen injection (*n* = 28 in each group; LRK Logrank test). Body weight of the 13 *Taf4*^*IEC*^ mice surviving up to the end of the experiment and of 13 mice of the Ctrl group at day 0 and day 120 after Tamoxifen administration. ns not significant; *p = 0.01; **p < 0.02; ****p < 0.0001. Boxes extend from the 25th–75th percentile and whiskers represent mean to max. **C** Immunodetection of the indicated proteins in the ileum of *Taf4*^*IEC*^ and control mice 10 days after Tamoxifen injection. Bars are 100 µm for Alpi, Aldob, Muc2 and Chga, and 50 µm for Dclk1, Olfm4, Lyz and Sox9. **D** Ki67 immunostaining and cell counts in the ileum of *Taf4*^*ΔIEC*^ and control mice. Bars are 100 µm. Boxes extend from the 25th–75th percentile and whiskers represent mean to max. *p < 0.05. **E** Immunofluorescent staining of phospho-Erk1/2 (red) and β-catenin (green) in *Taf4*^*IEC*^ and control mice. Bars are 100 µm. **F** BrdU detection in the jejunal mucosa of *Taf4*^*IEC*^ and control mice at the indicated days after the single injection of BrdU. Bars are 200 µm.

*Taf4* was inactivated in the adult gut epithelium of *Taf4*^*lox/lox*^*::VilCreER*^*T2*^ (hereafter *Taf4*^*IEC*^) and control mice by Tamoxifen injection at the age of 2–3-month-old. *Taf4* inactivation did not alter the general morphology of the intestinal crypt-villus axis (Fig. 2A). However, a significant proportion of mice of the *Taf4*^*IEC*^ series (15/28, 54%) had to be sacrificed during the course of the experiment as they reached the ethical limit point of weight lost. At later time points, the remaining 13 mice showed significantly lower body weight compared to controls that gained body weight over the same period (Fig. 2B). Progressively, *Taf4*^*IEC*^ mice were less active and suffered from diarrhea. They exhibited a swollen cecum distended by gas and the whole intestine appeared mechanically fragile.

The gut epithelium of Tamoxifen-injected *Taf4*^*IEC*^ mice exhibited cellular alterations in both proliferation and differentiation compartments (Fig. 2C). In the villi, enterocytes showed lower Alpi activity but increased Aldolase levels. The number of Muc2-positive goblet cells was reduced and enteroendocrine cells expressed less Chga. Dclk1 labeling of tuft cells was also strongly reduced. In the crypts, Sox9 was unaltered whereas Lyz in Paneth cells and Olfm4 in SCs were reduced, but still expressed. Ephb2, another marker of SCs was also reduced, although less than Olfm4 (Supplementary Fig. 1A). Functionally, the overall reduction of differentiated cells and/or of their features were associated with increased cell proliferation visualized with Ki67, higher phospho-Erk1/2 staining in the proliferative compartment, and accelerated cell turnover assessed by BrdU pulse-chase labeling (Fig. 2D, F). There was also a tendency to increased apoptosis displayed by activated Caspase-3, whereas the expression patterns of transcription factors Cdx2, Hnf4α 1–6 and 7–9, and Hes1 were unaltered (Supplementary Fig. 2).

To strengthen the relationship between the loss of Taf4 protein in SCs and the defective differentiation of mature villi cells, *Taf4*^*lox/lox*^*::Lgr5-GFP-CreER*^*T2*^ mice (hereafter *Taf4*^*CBC*^) were generated to selectively inactivate *Taf4* in the CBCs. Adult mice were treated with Tamoxifen and analyzed 6 days later. As expected from the mosaic expression of the *Lgr5-GFP-CreER*^*T2*^ allele in CBCs, only few crypts exhibited Taf4 loss in SCs whereas it remained present in adjacent Paneth cells due to their slow turnover (Supplementary Fig. 1B). *Taf4*^*CBC*^ mice showed villi lined by Taf4-positive cells with few intermingled ribbons of Taf4-negative cells originating from the Taf4-depleted crypts. Taf4-negative cells in the villi exhibited less Alpi activity compared to adjacent Taf4-expressing cells, indicating that Taf4 inactivation in SCs compromised the terminal differentiation of their progeny migrating up the villi (Supplementary Fig. 1C).

Altogether, these results highlight that *Taf4* inactivation perturbs the dynamic homeostasis of the SC niche and transit amplifying cell compartment ultimately leading to impaired differentiation of both absorptive and secretory cell lineages.

### *Taf4* regulates proliferation and the immune/inflammatory microenvironment in fetal and adult intestine

**Fig. 3:**
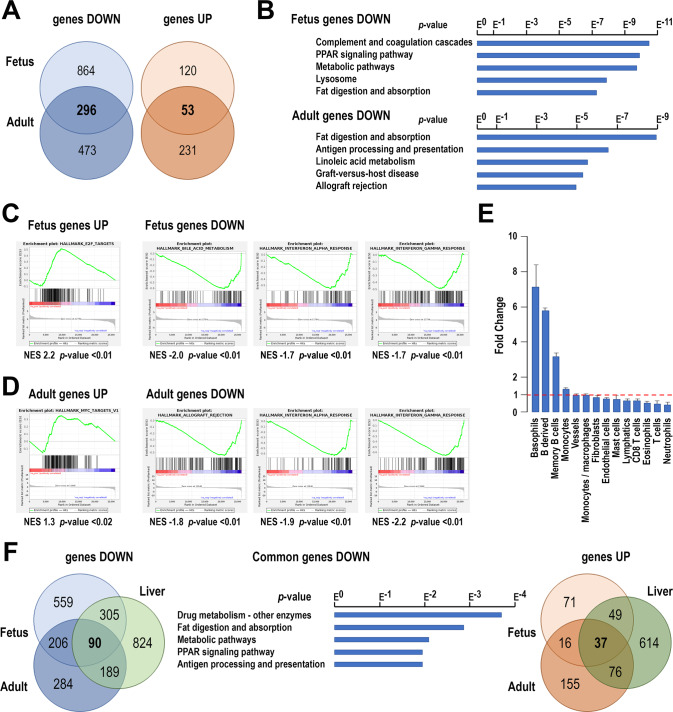
Gene expression changes after *Taf4* inactivation. **A** Venn diagrams representing the down- and up-regulated genes in E17.5 *Taf4*^*IEndoC*^ fetuses and adult *Taf4*^*IEC*^ mice compared to their respective controls. **B** KEGG ontology enrichment is shown for the downregulated genes in fetuses and adults, ordered according to the *p* value. **C** GSEA analysis of the up-regulated and downregulated genes in fetuses identifying respectively hallmarks for E2F targets and for bile acid metabolism, and IFNα and IFNγ response. **D** GSEA analysis of the up-regulated and downregulated genes in adults identifying respectively hallmarks for Myc targets and for allograft rejection, and IFNα and IFNγ response. **E** Stromal cell population evaluation from RNA-seq data using the MCP method, expressed as the proportion of each cell type in *Taf4*^*IEC*^ mice compared to controls. **F** Venn diagrams of the down-regulated genes (left) and up-regulated genes (right) enriched in common in the intestine of E17.5 *Taf4*^*IEndoC*^ fetuses and adult *Taf4*^*IEC*^ mice, and in the *Taf4*-null liver of 12 days suckling mice. Middle: KEGG ontology enrichment of the 90 genes down-regulated in common in the fetal and adult intestine and in the liver of *Taf4*-deficient mice.

The effect of *Taf4* inactivation on gene expression was investigated by RNA-seq in the intestine of E17.5 *Taf4*^*IEndoC*^ fetus vs. control littermates (Supplementary Table 1), and in the ileum of adult *Taf4*^*IEC*^ vs. control *Taf4*^*lox/lox*^ mice 10 days after Tamoxifen administration (Supplementary Table 2). Comparing the transcriptional changes resulting from *Taf4* inactivation in E17.5 *Taf4*^*IEndoC*^ and adult *Taf4*^*IEC*^ mice revealed a higher number of down-regulated than up-regulated genes at both stages (respectively 1160 vs. 173 in fetuses and 769 vs. 284 in adults, (|log_2_(FC) | > 1, *p* value < 0.05) (Fig. 3A). At E17.5, KEGG and GSEA analyses showed that down-regulated genes were enriched in terms associated with metabolism, reflecting the alteration of functional epithelial cell differentiation (Fig. 3B, C). GSEA analyses of up-regulated genes revealed enrichment in terms associated with increased proliferation (Fig. 3C). Loss of *Taf4* in the adult intestine corroborated and extended the immune-histological data, with many genes involved in enterocyte functions down-regulated, including those coding for digestive enzymes (Alpi, Anpep, Treh), fatty acid binding proteins (Fabp2, -6, -7), and more than 40 soluble carrier family members among which the transporters/cotransporters of Na/Glucose (Slc5a11/Sglt6, Slc5a12/Smct2), oligopeptides (Slc15a1/Ppept1), short-chain fatty acids (Slc16a3), amino acids (Slc7a8, Slc7a9, Slc7a15, Slc38a3) and folate (Slc46a1, Slc46a3) (Supplementary Table 2). However, the α-glucosidase genes *Sis* and *Mgam* and the aldolase gene *Aldob* were upregulated. Goblet cell mucin genes *Muc4* and *Muc20* were decreased, along with enteroendocrine genes encoding precursors and regulatory peptides (*Chga*, *Chgb*, *Cck*, *Gip*, *Pyy*, *Sct* and *Sst*) as well as *Dclk1* and *Pou2f3* for tuft cells. KEGG and GSEA analysis confirmed enrichment of the down-regulated genes in different aspects of metabolism (Fig. 3B, D), whereas GSEA analysis of the up-regulated genes showed enrichment in cell proliferation (Fig. 3D). In addition, altered gene expression was observed in the SC niche, as exemplified by reduced expression of CBC signature genes (*Agr3*, *Ciita*, *Esrrg*, *Fras1*, *H2-Eb1*, *Hk2*, *Lect2*, *Olfm4*, *Rdh16*, *Sdsl*, *Sectm1b*, *Tifa*, *Tnfsf10*, *Tns4*, *Vnn1*) [6] as well as Paneth cell genes (*Defa26*, *Dafa-rs1*, *Defb1*, *Defb37*, *Lyz1*, *Mmp7*). Thus, *Taf4* inactivation perturbed the SC compartment and terminal differentiation of all mature epithelial cell types migrating along the villi.

A subset of genes was de-regulated in a similar manner in both the embryonic and adult contexts (Fig. 3A), sharing enrichment of several GSEA and KEGG ontology pathways notably associated with increased proliferation for the up-regulated genes and inflammation/immune functions including allograft rejection and the interferon (IFN) alpha and IFN gamma pathways for the down-regulated genes. Murine Microenvironment Cell Population (MCP [7]) counter analyses of the RNA-seq data further showed a modification of the immune microenvironment with increased number of basophils and B cells and reduced T cells in the *Taf4*-mutant adult intestinal mucosa (Fig. 3E). *Taf4* inactivation thus had cell-autonomous effects on epithelial cells, but also a non-cell-autonomous impact on the mucosal microenvironment.

We previously reported that *Taf4* inactivation in endoderm-derived neonatal hepatocytes also upset metabolic pathways [4]. Among the down-regulated genes in hepatocytes, 395 were shared with the fetal gut and 279 with the adult gut, 90 of them being affected in all 3 contexts (Fig. 3F). KEGG pathway analysis linked these 90 genes to several aspects of metabolism, complement function and chemical carcinogenesis (Fig. 3F).

### *Taf4* inactivation in the adult gut modifies the microbiota and enhances the inflammatory response

Along with digestive and metabolic functions, the gut epithelium plays an important barrier function at the interface between the luminal microbiota and the stroma. Diarrhea and the swollen cecum after *Taf4* loss suggested changes in the microbiota. Comparison of the bacterial composition within the cecum of Tamoxifen-treated *Taf4*^*IEC*^ and control *Taf4*^*lox/lox*^ mice 1 month after *Taf4* inactivation revealed reduced α-diversity with fewer operational taxonomic units (OTUs) in mutants than in controls (Supplementary Fig. 3A). The microbiota of *Taf4*^*IEC*^ mice showed higher abundance of *Helicobacteriaceae*, *Bifidobacteriaceae* and *Deferribacteraceae* families but reduction of *Porpyromonadaceae*. At genus level, seven genera displayed differential levels: *Bifidobacterium*, *Clostridium XIVb* and *XVIII*, *Helicobacter*, *Mucispirillum* and *Olsenella* were significantly more abundant in mutants, whereas controls harbored more *Marvinbryantia*. OTUs included in *Helicobacter* genera were close to *H. typhlonius* (100%), *H. ganmani* (98%) and an uncultured species (JRPC—100%), and those included into *Bifidobacterium* genera close to *B. pseudolongum* (98%) and *B. animalis* (100%); *M schaedleri* (100%) was the main strain among *Mucispirillum* genera (Supplementary Fig. 3A). Two genera found in higher abundance in the cecum of *Taf4* deficient mice, *Helicobacter* and *Olsenella*, usually colonizers of the upper part of the digestive tract, are considered as detrimental for host health. *Bifidobacterium*, *Mucispirillum* and *Clostridium XIVb* and *XVIII*, also in higher proportion in *Taf4*-deficient mice, are commensals of the gut ecosystem with a metabolism oriented toward SCFA production, whereas *Marvinbryantia*, preferentially found associated with small intestinal microbiota and including species that use cellulose to produce acetate [8], are reduced in *Taf4*-deficient mice. Thus, *Taf4* inactivation in the intestinal epithelium impacts the composition of the gut microbiota by reducing its diversity and favoring the presence of microbes from the upper part of the digestive tract in the distal part, while maintaining SCFA-producing bacteria whose activity should support higher cell proliferation rate of colonocytes. Interestingly, the microbiota changes triggered by *Taf4* invalidation were accompanied by increased paracellular permeability of the epithelium measured in vivo by FITC-Dextran luminal-to-blood transfer, whereas transcellular permeability measured with D-Xylose remained unchanged (Supplementary Fig. 3B). It is worth noting that decreased microbiota diversity is observed in several pathological contexts where the mucosal barrier integrity/functionality is altered [9].

Considering the changes in epithelial barrier activity, microbiota composition and stromal cell composition induced by *Taf4* loss, mice were challenged with a pro-inflammatory stimulation. For this purpose, *Taf4*^*IEC*^ and control *Taf4*^*lox/lox*^ mice (*n* = 5 in each group) were treated with Tamoxifen and 12 days later were given 2% DSS in drinking water for 5 days before returning to tap water for 3 days. Two *Taf4*^*IEC*^ mice exhibited traces of blood in the rectum already before DSS treatment, and 2 mice of this group were euthanized in the 2 days after the end of DSS treatment because they had reached the ethical limit point. Survey of the mice during the course of the experiment revealed a significantly higher clinical score in *Taf4*-deficient mice compared to controls (*p* = 0.0009) (Supplementary Fig. 3C), while the length of the colon was also shorter in mutants at the end of experiment (5.6 cm vs. 8.1 cm, *p* = 0.012). Histological examination showed a nearly normal structure with a regular glandular organization of the distal colon of control mice after the 2% DSS treatment, whereas *Taf4*-inactivated mice exhibited a severely altered colonic mucosa with edema, large areas of immune cell clots, and an irregular single epithelial layer with few remnant glands (Supplementary Fig. 3D). The mutant epithelium showed strongly reduced cell proliferation, high proportion of apoptotic cells labeled with activated Caspase-3 and homogeneous expression of Ephb2 (Supplementary Fig. 3D). These observations demonstrated exacerbated sensitivity of *Taf4*-deficient mice to acute inflammation.

### Impact of *Taf4* inactivation on enteroid morphogenesis

**Fig. 4:**
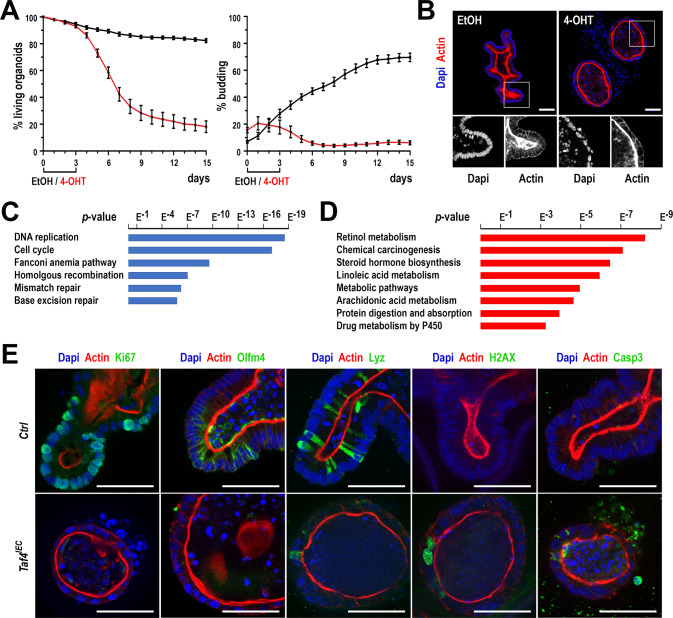
Effect of *Taf4* inactivation on enteroid morphogenesis. **A** Survival and budding activity after early *Taf4* gene inactivation. *Taf4*^*IEC*^ enteroids were plated and treated 2 h later with 4-OHT in EtOH (red line) or EtOH alone (black line) for 3 days. In total, 50–100 3D structures were counted at each time point. **B** Morphology of the *Taf4*^*IEC*^ enteroids at day 5 of culture, that is 2 days after treatment with EtOH (left) or 4-OHT (right). Boxed regions are enlarged below. Nuclei are stained with Dapi (blue) and the actin network with Phalloidin (red). Bars are 50 µm. **C** Top 6 enriched KEGG pathways in downregulated genes in 4-OHT treated *Taf4*^*IEC*^ vs. *Taf4*^*lox/lox*^ enteroids. **D** Top 8 enriched KEGG pathways in upregulated genes in 4-OHT treated *Taf4*^*IEC*^ vs. *Taf4*^*lox/lox*^ enteroids. **E** Immunofluorescence detection of the indicated proteins in *Taf4*^*lox/lox*^ (Ctrl) and *Taf4*^*IEC*^ enteroids treated with 4-OHT at day 5 of culture (2 days after treatment with 4-OHT). Bars are 50 µm.

To better understand the cell autonomous effects of *Taf4* loss in the intestinal epithelium cells, we developed crypt-derived 3D enteroids and inactivated *Taf4* ex vivo [10]. For this purpose, enteroids were established from the ileum of Tamoxifen-free *Taf4*^*IEC*^ and control *Taf4*^*lox/lox*^ mice. As expected, enteroids of both genotypes cultured in standard medium generated typical 3D structures with crypt-like buddings lined by a single polarized epithelium. An analogous situation was observed with *Taf4*^*lox/lox*^ enteroids treated with 4-Hydroxy-Tamoxifen (4-OHT) during the first 3 days of culture. In contrast, 4-OHT-treated *Taf4*^*IEC*^ enteroids resulted in altered 3D structures characterized by a flat cuboidal epithelium, reduced budding outgrowths, and accumulation of cell debris and DNA within the lumen and even outside the spheroids, as illustrated at day 5 of culture (Fig. 4A, B). Ultimately, these disorganized cystic enteroids degenerated, indicating compromised morphogenesis and survival upon Taf4 loss.

The consequence of *Taf4* inactivation on enteroid gene expression was investigated by RNA-seq performed after 3 days of 4-OHT treatment, to capture the most direct effects of *Taf4* loss at a stage when the cellular phenotype was still mild. Compared to control 4-OHT-treated *Taf4*^*lox/lox*^ enteroids, treated *Taf4*^*IEC*^ enteroids showed 3269 deregulated genes (Supplementary Table 3). KEGG annotation of the 1623 down-regulated genes identified DNA replication/cell cycle and DNA repair as the major pathways affected by *Taf4* loss (Fig. 4C). Specifically, 182 of the 510 genes of the intestinal SC signature [6] were down-regulated, including receptor genes present on CBCs: *Fzd7*, *Fzd2*, *Lgr5*, *Notch1* and *Tnfrsf19*. Genes encoding ligands of these receptors, expressed by Paneth cells in the epithelial SC niche, were also down-regulated: *Wnt3*, *Wnt5a*, *Wnt9b*, *Dll3*, *Jag2*, *Egfl8*. Expression of the *Cdx2* homeobox gene, that dictates intestinal identity in SCs, was perturbed as well as genes encoding differentiation markers of mature intestinal cell types, among which *Defa17/20/21/24/26* and *Mmp7* for Paneth cells, *Muc4/20* and *Tff2* for goblet cells, *Chga/b* and *Cck* for enteroendocrine cells, and *Aldoa* and *Fabp5* for enterocytes. However, other marker genes were upregulated such as *Cdhr2/5*, *Dpp4*, *Fabp1/2/6*, *Mgam*, *Sis* and *Vill*. KEGG analysis of the upregulated genes identified pathways related to chemical carcinogenesis and metabolic processes (Fig. 4D).

Immunofluorescence at day 5, that is 2 days after the end of 4-OHT treatment, corroborated the transcriptomic results (Fig. 4E). Compared to controls, *Taf4* loss led to a strong reduction of the cell proliferation marker Ki67. The decline in cell proliferation was associated with loss of the SC marker Olfm4 and reduction of Lysozyme in Paneth cells. In addition, considering the number of downregulated genes involved in DNA replication and repair, including *Kdm2b*, *Ppar1* and *Timeless* that signal DNA damage response [11], a strong punctate staining of γH2AX was observed selectively in 4-OHT-treated *Taf4*^*IEC*^ enteroids. In line with this, activated Caspase-3-labeled apoptotic cells were detected in *Taf4*^*IEC*^ but not in control *Taf4*^*lox/lox*^ enteroids.

Thus, *Taf4* loss alters the SC gene expression program and triggers defects in DNA integrity and replication leading to cell apoptosis ultimately eliciting enteroid degeneration and death.

### Impact of *Taf4* inactivation on enteroid maintenance

**Fig. 5:**
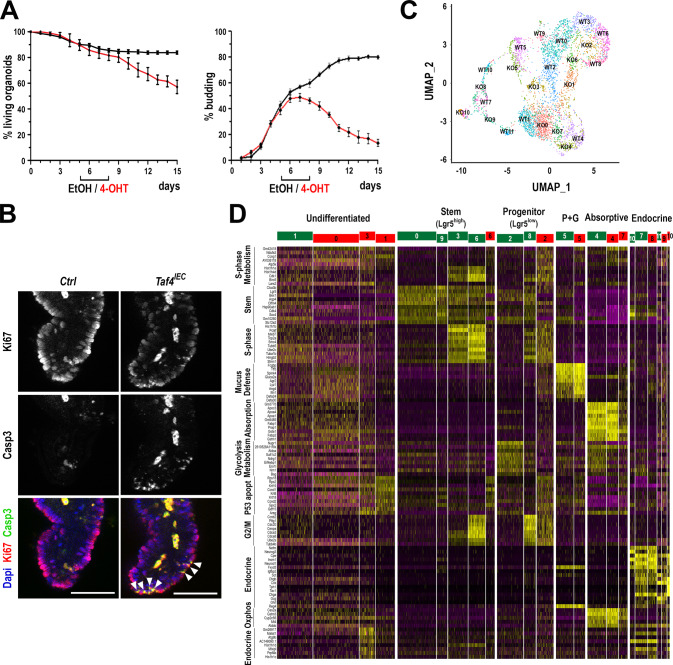
Effect of *Taf4* inactivation on enteroid homeostasis. **A** Survival and budding activity after late *Taf4* gene inactivation. *Taf4*^*IEC*^ enteroids were plated and treated with 4-OHT (red line) or EtOH (black line) at days 5 to 7 of culture. In total, 50–100 3D structures were counted at each time point. **B** Immunodetection of proliferating cells (Ki67) and apoptotic bodies (activated Caspase-3) at day 8 of culture in 4-OHT-treated *Taf4*^*IEC*^ vs. *Taf4*^*lox/lox*^ (Ctrl) enteroids. White arrowheads show costaining. Bars are 50 µm. **C** Uniform manifold approximation and projection (UMAP) clustering from scRNA-seq analyses in 4-OHT-treated *Taf4*^*IEC*^ and *Taf4*^*lox/lox*^ enteroids at day 8 of culture. Cell clusters identified in *Taf4*^*lox/lox*^ and *Taf4*^*IEC*^ enteroids were labeled WT0 to WT11 and KO0 to K010, respectively. **D** Heatmap of the top markers identified by scRNA-seq in the WT0 to WT11 (green) and KO0 to K010 (red) cell clusters. Absorptive: enterocytes; P + G: Paneth and goblet cells, Endocrine: enteroendocrine cells.

Having demonstrated the important role of Taf4 on enteroid morphogenesis, we asked if it was required for enteroid maintenance, when buddings were already formed with SCs in their niche. For this purpose, enteroids were grown for 5 days in the absence of 4-OHT that was then added to the culture medium for 3 days. Under these conditions, *Taf4*^*IEC*^ enteroids progressively lost buddings generated during the first 5 days of culture, stopped growing but remained viable up to day 15, although they could no longer be passaged (Fig. 5A). Immunofluorescence for Ki67 demonstrated reduced cell proliferation in *Taf4*^*IEC*^ enteroids (Fig. 5B). Moreover, the presence of apoptotic bodies at the level of Ki67-positive cells was seen by activated Caspase-3 immunodetection, suggesting that proliferative cells enter apoptosis in the absence of Taf4.

To decipher the impact of *Taf4* loss on the different cell populations that make up the enteroid at this stage, we performed single-cell (sc) RNA-seq at day 8 of culture. UMAP representation defined a collection of cell populations from both the 4-OHT-treated control and *Taf4*^*IEC*^ enteroids (Fig. 5C) that displayed distinctive expression signatures allowing their identification (Fig. 5D and see Supplementary Datasets 4–8). Based on the level of Lgr5 and Olfm4 expression [12–14], control enteroids exhibited 4 Lgr5^high^ Olfm4^high^ SC clusters, two of them in non-cycling state (WT0, WT9) and two in S and S/G2/M states (WT3, WT6), together with two Lgr5^low^ progenitor clusters, one in S/G2/M state still expressing Olfm4 (WT8), and the other displaying a glycolytic signature without Olfm4 (WT2). Compared to the clusters identified in controls, *Taf4*^*IEC*^ enteroids revealed a strong reduction of Lgr5^high^ SC cells that additionally displayed a p53/apoptotic signature (cluster KO6), and a reduction of Lgr5^low^ progenitors also exhibiting a p53/apoptotic signature (cluster KO2). In addition, *Taf4*-inactivated enteroids showed an increase in cells with no clearly defined identity (undifferentiated; clusters KO0, ΚΟ1, ΚΟ3 vs. WT1), two of them with a p53/apoptotic signature (KO1, KO3). At this stage of culture, there was no major effect on Paneth and goblet cells (cluster KO5 vs. WT5), whereas a poorly differentiated sub-population of enterocytes emerged (clusters KO4, KO7 vs. WT4). The pattern of enteroendocrine cells was also perturbed, characterized by a reduction of precursor cells and the presence of poorly differentiated cells (clusters KO8, KO9, KO10 vs. WT7, WT10, WT11).

*Taf4* loss therefore not only affects enteroid morphogenesis and budding formation, but it also compromises the maintenance of buddings through a strong reduction in stem and progenitor cell populations.

### Chromatin conformation changes after *Taf4* inactivation

**Fig. 6:**
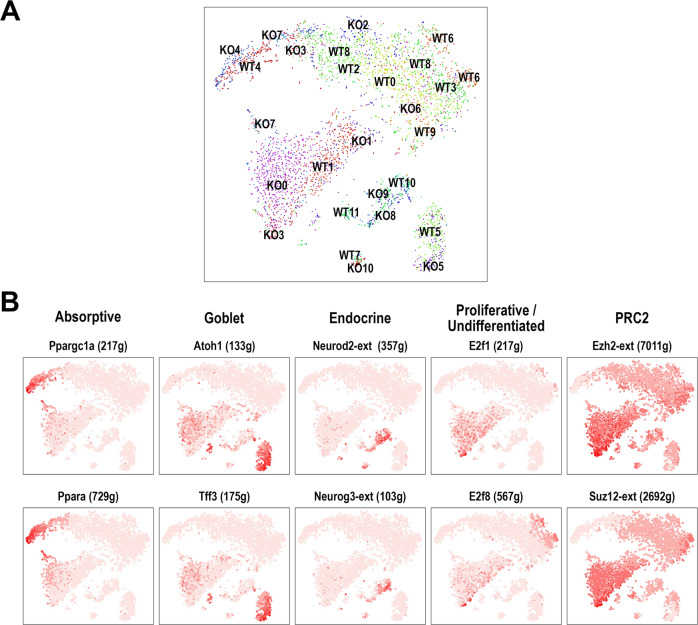
Regulons changes associated with Taf4 loss. **A** SCENIC-UMAP of the cell clusters identified by scRNA-seq in 4-OHT-treated *Taf4*^*lox/lox*^ (WT) and *Taf4*^*IEC*^ (KO) enteroids. **B** Uncertainty-Aware Face Clustering (AUC) of representative highly active regulons in the indicated cell clusters identified by SCENIC in *Taf4*^*lox/lox*^ enteroids.

ATAC-seq performed after early *Taf4* inactivation at day 3 of 4-OHT treatment in *Taf4*^*IEC*^ vs. *Taf4*^*lox/lox*^ enteroids provided a broad picture of chromatin remodeling linked to the loss of Taf4 (Supplementary Fig. 4A and Supplementary Table 9). From 118,500 non-redundant peaks present in control and *Taf4*-inactivated enteroids, seq-MINER analysis revealed a cluster of 6874 peaks that were diminished (cluster C7-4) and two clusters of respectively 3798 (cluster 8-1) and 5021 augmented peaks (cluster 8-2) (Supplementary Fig. 4B). DNA-binding motif analysis in these clusters, and in silico footprinting of differentially accessible sites [15] showed a specific enrichment of DNA-binding sites for nuclear receptors (Hnf4α/γ, Pparα/γ, RXRα/γ, Nr2c2, Nr2f6) and Jun- or Fos-containing AP1 dimers in control enteroids and for Zinc finger transcription factors (Gata, Sp2/4, Ctcf) in the Taf4-mutant enteroids (Supplementary Fig. 5C, D). Interestingly, diminished and/or augmented peaks were linked to 585 of the 1623 downregulated genes after Taf4 loss. Gene ontology associated these 585 downregulated genes to cell cycle and division, DNA replication and cell response to DNA damage (Fig. 6E). Among them, 75 were contained in the set of 182 genes of the SC signature downregulated after Taf4 loss, including *Axin2*, *Fzd7*, *Lgr5*, *Notch1* and *Olfm4*.

### Rescue of *Taf4*-depleted enteroids by Polycomb inhibition

*Taf4* inactivation in enteroids led to loss of the SC and transit amplifying cell compartments with a concomitant increase in undifferentiated cells, accompanied by chromatin remodeling and decreased expression of many SC signature genes consistent with transcriptional reprogramming. The degeneration of *Taf4*-inactivated enteroids indicated the failure of known rescue mechanisms ex vivo. Thus, the mobilization of “+4” or “revival” cells should be defective despite the increased expression of regenerative markers such as *Tert*, *Kr19* and *Clu* [16–18], as well as the YAP-dependent reprogramming of SCs, even if some genes of the YAP pathway for intestinal repair were modified, including the decrease of *Reg3a*, *Olfm4*, *Wnt3*, *Aqp4, Lgr5* and *Axin2* and the increase of *Areg*, *Il1rn* and *Msln* [19] (Supplementary Table 2). In addition, *Ascl2* expression was markedly reduced in *Taf4*-deficient enteroids preventing *Ascl2*-dependent replenishment of the SC compartment from progenitors [20] (Supplementary Table 2). To address the mechanism underlying enteroid degeneration, we performed single-cell regulatory network inference and clustering (SCENIC) analysis on the control and *Taf4*-inactivated cells (Fig. 6A). Several of the cell populations were strongly marked by activity of factors already shown to be important for their identity and/or specification (Fig. 6B): Atoh1 and Tff3 for Goblet cells, Neurod2 and Neurog3 for enteroendocrine cells, and nuclear receptors such as Ppara and the master regulator of metabolism Ppargc1a for enterocytes in agreement with their high OXPHOS signature. The undifferentiated cells and transit amplifying cells were also marked by activity of E2f factors consistent with their cell cycle signatures. Nevertheless, SCENIC did not identify transcription factor regulons strongly active in the SCs. Instead, we noted that the SCs displayed low Ezh2 and Suz12 activity that was strongly increased in the control and *Taf4*-inactivated undifferentiated cells suggesting that increased PRC2 complex activity played a role in suppressing SC identity. As the undifferentiated cell population increased at the expense of the SC compartment, we thus postulated that Taf4 loss promoted PRC2 activity leading to suppression of SC identity thereby favoring the undifferentiated state.

**Fig. 7:**
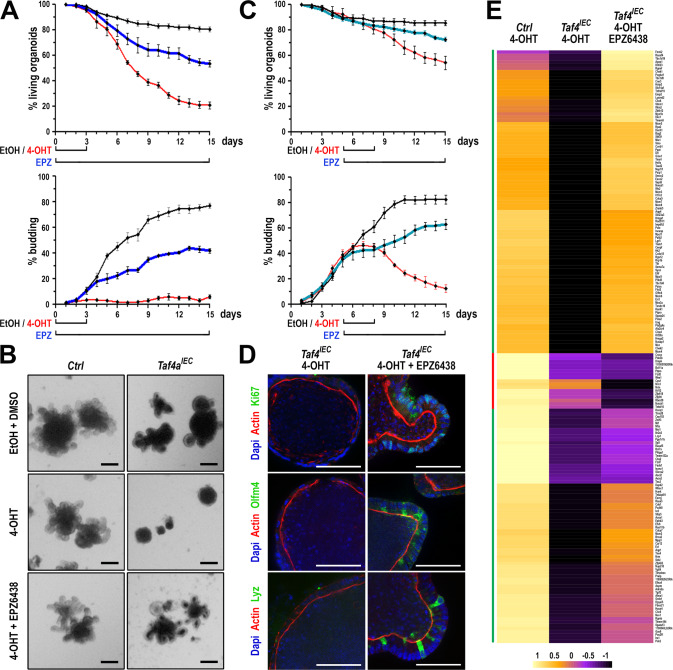
Rescue of *Taf4* inactivated enteroids by Polycomb complex inhibition. **A** Survival and budding activity in *Taf4*^*IEC*^ enteroids treated with EtOH + DMSO (black line), with 4-OHT (2–72 h after plating), or 4-OHT (2–72 h after plating) + EPZ6438 (2 h to 15 days after plating) (red line). In total, 50-100 3D structures were counted at each time point. **B** Morphology at day 15 of culture of *Taf4*^*lox/lox*^ (ctrl) and *Taf4*^*IEC*^ enteroids treated with EtOH + DMSO, or 4-OHT (2–72 h after plating), or 4-OHT (2–72 h after plating) + EPZ6438 (2 h to 15 days after plating). Bars are 200 µm. **C** Survival and budding activity in *Taf4*^*IEC*^ enteroids treated with EtOH + DMSO (black line), with 4-OHT (days 5-8 after plating) (red line), or with 4-OHT (days 5-8 after plating) + EPZ6438 (days 5-15 after plating) (blue line). In total, 50–100 3D structures were counted at each time point. **D** Immunostaining of the indicated proteins in *Taf4*^*IEC*^ enteroids treated 4-OHT or with 4-OHT + EPZ6438 at day 15 of culture. Bars are 50 µm. **E** Heatmap of the 182 genes of the stem cell signature that are downregulated by Taf4 loss in 4-OHT treated *Taf4*^*IEC*^ enteroids compared to treated *Taf4*^*lox/lox*^ control enteroids, and restored by treatment with the Ezh2 inhibitor EPZ6438.

To test this idea, 4-OHT-treated *Taf4*^*IEC*^ and control *Taf4*^*lox/lox*^ enteroids were treated with EPZ6438, a selective inhibitor of the Ezh2 histone methyltransferase of PRC2. EPZ6438 was added at the same time as 4-OHT and maintained up to day 15. When *Taf4* was inactivated early after plating, EPZ6438 promoted survival and budding in *Taf4*^*IEC*^ enteroids (Fig. 7A, B and Supplementary Fig. 5A). EPZ6438 treatment also rescued budding degeneration by late *Taf4* inactivation at day 5 of culture (Fig. 7C and Supplementary Fig. 5B). Noteworthy, immunofluorescence demonstrated that the buddings restored by EPZ6438 in *Taf4*-inactivated *Taf4*^*IEC*^ enteroids treated with 4-OHT were populated with proliferating cells (Ki67), SCs (Olfm4) and Paneth cells (Lyz) (Fig. 7D). This result was further extended by RNA-seq (Supplementary Table 10). Indeed, among the 1623 genes downregulated by *Taf4* inactivation, the expression of 1146 (70.6%) was restored by adding EPZ6438 (*p* < 0.05), of which 949 (58.5%) with a Fold Change > 2. Similarly, among the 1646 genes upregulated after Taf4 loss, 1166 (70.8%) were decreased by EPZ6438, of which 947 (57.5%) with a (|log_2_(FC) | > 1. Importantly, expression of 115 of the 182 genes of the SC signature down-regulated by the loss of Taf4 were restored by EPZ6438 (Fig. 7E).

To determine if *Taf4* loss also affected PRC2 function in the developing intestinal epithelium and adult mucosa, we performed immunohistochemistry for the PRC2-dependent histone mark H3K27me3 (Supplementary Fig. 6). Control E18.5 embryos showed low H3K27me3 staining in the intervilli regions and progressively increasing levels along the villi. Similarly, adults exhibited increasing H3K27me3 levels along the villi, with few cells labeling at the bottom of the crypts. In contrast, H3K27me3 staining was strong in all of the cells making up the flat developing epithelium in *Taf4*^*IEndoC*^ embryos consistent with the lack of SC emergence. In adult *Taf4*^*IEC*^ mice, increased H3K27me3 levels were seen in both the villi and most strikingly in cells at the crypt bottom compared to controls, in line with reduced SC activity. Thus, consistent with the stimulation of PRC2 activity after *Taf4* inactivation in enteroids, *Taf4* gene inactivation during intestinal morphogenesis in embryos and in adult intestinal homeostasis resulted in elevated levels of PRC2-deposited H3K27me3 and impaired SC development or function.

### *Taf4* inactivation enhances *Apc*-driven tumorigenesis

**Fig. 8:**
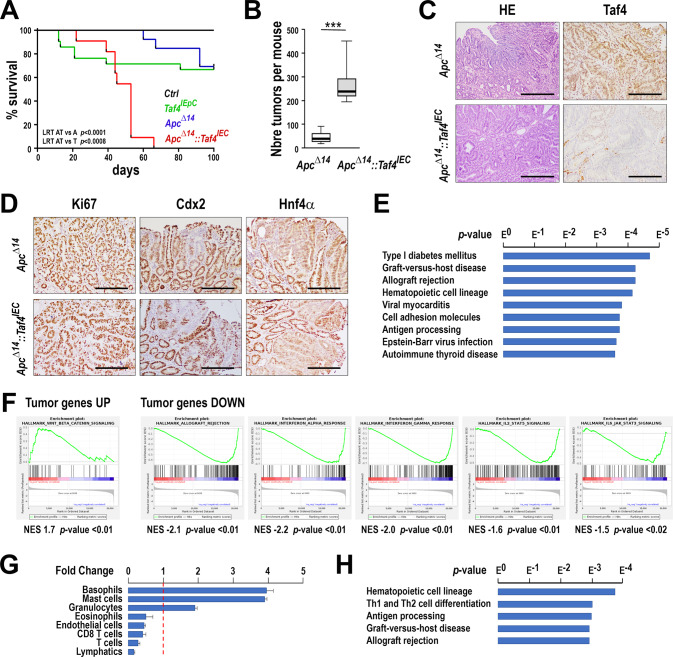
Impact of *Taf4* inactivation on intestinal tumor development. **A** Overall survival of *Apc*^*+/Δ14*^*::Taf4*^*IEC*^, *Apc*^*+/Δ14*^, *Taf4*^*IEC*^ and *Taf4*^*lox/lox*^ (Ctrl) males after Tamoxifen injection; *n* > 10 for each genotype. LRT AT vs. A: Logrank test between *Apc*^*+/Δ14*^*::Taf4*^*IEC*^ and *Apc*^*+/Δ14*^ mice; LRT AT vs. T: Logrank test between *Apc*^*+/Δ14*^*::Taf4*^*IEC*^ and *Taf4*^*IEC*^ mice. **B** Tumor number in the small intestine of *Apc*^*+/Δ14*^*::Taf4*^*IEC*^ and *Apc*^*+/Δ14*^ mice. ***p < 0.0001. **C** Histology (HE) and immunodetection of Taf4 protein in ileal tumors of *Apc*^*+/Δ14*^*::Taf4*^*IEC*^ and *Apc*^*+/Δ14*^ mice. Bars are 400 µm for HE and 200 µm for Taf4. **D** Immunohistochemical detection of Ki67, Cdx2 and Hnf4α in *Apc*^*+/Δ14*^*::Taf4*^*IEC*^ and *Apc*^*+/Δ14*^ tumors. Bars are 200 µm. **E** KEGG ontology enrichment is shown for the 699 downregulated genes in *Apc*^*+/Δ14*^*::Taf4*^*IEC*^ vs. *Apc*^*+/Δ14*^ mice and ordered according to the *p* value. **F** GSEA analysis of the up-regulated and downregulated genes in *Apc*^*+/Δ14*^*::Taf4*^*IEC*^ vs. *Apc*^*+/Δ14*^ mice identifying respectively hallmarks for Wnt/β-catenin signaling and for allograft rejection and IFNα, IFNγ, IL2/STAT5 and IL6/STAT3 signaling. **G** Stromal cell population evaluation from RNA-seq data using the MCP method, expressed as the proportion of each cell type in *Apc*^*+/Δ14*^*::Taf4*^*IEC*^ compared to *Apc*^*+/Δ14*^ mice. **H** KEGG ontology enrichment of the 247 downregulated genes in common between *Apc*^*+/Δ14*^*::Taf4*^*IEC*^ vs. *Apc*^*+/Δ14*^ mice and *Taf4*^*IEC*^ vs. wild type mice.

Since *Taf4* inactivation led to increased PRC2 activity in crypt SCs, that in enteroids resulted in the increased pool of undifferentiated cells, we asked if this alteration may affect intestinal tumorigenesis in the tumor prone model of *Apc*^*+/Δ14*^ mice. In a cohort of males (*n* > 10 in each group), *Taf4* loss in *Taf4*^*IEC*^*::Apc*^*+/Δ14*^ mice reduced overall survival and aggravated tumor burden compared to *Apc*^*+/Δ14*^ mice (Fig. 8A, B). *Taf4*^*IEC*^*::Apc*^*+/Δ14*^ tumors were histologically similar to those of *Apc*^*+/Δ14*^ mice (Fig. 8C), and exhibited a comparable distribution of proliferative cells labeled with Ki67, and a similar intratumor heterogeneity as illustrated by the patterns of Cdx2 and Hnf4α (Fig. 8D). RNA-seq of the tumors showed 699 down-regulated genes and 122 up-regulated genes upon *Taf4* inactivation (Supplementary Table 11). GSEA and KEGG analyses revealed a strong decrease in terms related to allograft-rejection and IFNα, IFNγ, IL2 / STAT5 and IL3 / STAT3 pathways together with increased of Wnt/β-catenin signaling (Fig. 8E, F). The down-regulation of these immune/inflammatory pathways was associated with an altered tumor immune-microenvironment. In *Taf4* depleted tumors, MCP counter showed an increase in Mast cells/Basophils and granulocytes, but a decrease of T cells amongst which CD8+ cytotoxic T lymphocytes (Fig. 8G), supported by reduced levels of their key markers Cd8a, Pdcd1, Tigit, and Lag3 (Supplementary Table 11). The striking reduction of the interferon / inflammation pathways and T cells in tumors mirrored the changes already seen in the normal gut mucosa. Indeed, more than 33% (247/699) of the down-regulated genes in tumors were also down-regulated in the non-tumoral mucosa upon *Taf4* inactivation (compare Supplementary Tables 2 and 11), and these 247 genes were associated with KEGG pathways designating hematopoietic cell lineages, Th1 and Th2 cell differentiation, antigen processing, graft-versus-host disease, and allograft rejection (Fig. 8H). *Taf4* loss both in non-tumoral gut mucosa and in intestinal tumors therefore led to an altered immune environment. In particular, both situations were characterized by reduced gene expression of *Cd7*, a marker of mature CD8+ T cells, of *Cd8a* itself, of the granzymes *Gzma* and *Gzmb* and of *Prf1* for cytotoxic T lymphocytes, together with the repression of the IFN gamma pathway, a major regulator of colon tumor immunity [21]. Moreover, expression of cytokine Cxcl11 that attracts cytotoxic T lymphocytes [22] was reduced, whereas expression of Interleukin-17 receptor (Il17Rb) was increased. The Il17-IL17Rb axis is known to inhibit cytotoxic T lymphocyte recruitment in mouse models of intestinal cancer [23, 24]. Together these results revealed a novel facet of Taf4 function in influencing the immune microenvironment in both normal mucosal tissue and in the tumor context.

## Discussion

### Taf4 is a critical regulator of development and homeostasis of the intestinal epithelium

This study defines the important role played by the Taf4 component of TFIID in intestinal development and homeostasis. Taf4 loss had not only cell-autonomous effects, but also affected gut permeability, the immune microenvironment and the composition of the luminal microbiota ultimately impacting the response to pro-inflammatory and pro-tumoral stimuli. Taf4 was crucial for the emergence of adult-type intestinal SCs during late embryogenesis and participated in their maintenance throughout adulthood together with differentiation of mature cells. The critical role of Taf4 in SCs was further confirmed in enteroids where its loss depleted the SC and transit amplifying compartments.

Although Taf4 is a subunit of a major component of the general transcription machinery, its loss of function affected only a subset of genes in the gut epithelium, in agreement with previous observations in liver, pancreas and epidermis [3–5]. We previously suggested that these limited effects on gene expression were in part explained by compensation by its paralog Taf4b [2, 25] that integrates and maintains the integrity of TFIID. This mechanism is also likely operative in the intestine epithelium since RNA-seq data in both fetal and adult intestine indicated a mild but significant increase of *Taf4b* expression upon *Taf4* loss.

In vivo, the defects observed upon *Taf4* loss demonstrated its involvement in the proper differentiation of mature cells of both absorptive and secretory lineages and in the control of the epithelial turnover and barrier function. Increased paracellular permeability did not reflect expression changes of cell-cell adhesion components, but correlated with a decrease of several integrin genes including *Itgb6* that encodes a subunit of αvβ6 participating in the barrier activity of intestinal epithelial cells [26]. Several of the genes downregulated in the developing and adult gut are shared with *Taf4*-deficient neonatal hepatocytes, including metabolic genes. In addition, ATAC-seq peaks diminished upon *Taf4* loss in intestinal enteroids were enriched in DNA-binding sites for Hnf4, a major regulator of metabolic genes previously shown to interact with Taf4 in hepatocytes [4]. Together, the data obtained in the intestine and liver suggest that Taf4 coordinates metabolic pathways by promoting Hnf4 binding and maintaining chromatin accessibility at key metabolic genes.

### A novel role of Taf4 in antagonizing PRC2 activity and regulating the immune-microenvironment

A key finding of our study is the novel role for Taf4 in antagonizing the activity of the PRC2 complex to maintain the SC gene expression program and the stem/progenitor cell compartment as highlighted in vivo during embryonic development and in the adult intestine, and ex vivo in enteroids. In the fetus, *Taf4* loss increased PRC2 activity in all cells lining the epithelium consistent with the impaired emergence of adult-type SCs, whereas in adults some features of the already established SCs were perturbed, for example diminished *Olfm4* expression, but a limited SC activity was preserved allowing cell turnover. The difference between fetuses/enteroids and adults suggests that extrinsic signals from the adult SC niche, likely insufficient during development and in enteroids, may partially compensate for Taf4 deficiency and maintain stemness, and thus that an integrated and fine-tuned epithelial-mesenchymal interaction is necessary to properly switch on the regenerative process when SCs are affected at the adult stage [27]. In line with this, transcriptomic data indicate that the level of *Wnt3*, an important Wnt family ligand for adult SCs [10, 28], was reduced in *Taf4*-deficient fetuses and enteroids, but not in the adult epithelium.

Our results reveal that inhibiting the methyltransferase activity of the Ezh2 component of PRC2 rescued enteroid survival, budding formation and cell proliferation, and restored the SC compartment with active SCs and supporting Paneth cells, all of which were compromised in the absence of Taf4. This was accompanied by the rescued expression of a large proportion of genes deregulated by *Taf4* loss, including genes of the intestinal SC signature. Previous studies showed how SWI/SNF recruitment by transcription factors to both enhancers and proximal promoters upon gene activation antagonizes PRC2 activity [29–31]. Our results extend this model by showing that the Taf4 subunit of TFIID, a key component of the promoter-bound pre-initiation complex and present at enhancers, also antagonizes the ability of PRC2 to supress the expression of the SC gene expression program. Yet, Taf4 is necessary to overcome the repression of this program by PRC2, but not for its expression per se that takes place in absence of Taf4 when Ezh2 is inhibited.

PRC1 and 2 are major regulators of epigenetic silencing in developmental processes and pathologies including cancer and inflammatory diseases [32, 33] and are involved in the control of intestinal crypt homeostasis and regeneration after damage [34, 35]. In enteroids, the SC and progenitor compartments were marked by low activity of Ezh2 and Suz12 in agreement with previous studies showing that PRC2 is essential for SC maintenance and proliferation [34–36]. In addition, differentiation of Paneth cells that provide ligands required for SC functions and emergence in enteroids [37] is also dependent on the level of Ezh2/PRC2 activity [38]. Finely tuned PRC2 activity in SCs and in the niche Paneth cells is therefore essential for proper homeostasis. Our observations in enteroids suggest that SCs exist in equilibrium with the undifferentiated population controlled by a competition between Taf4-driven expression of the SC program and its PRC2-mediated repression. Upon *Taf4* inactivation, this equilibrium is upset with increased PRC2 activity shutting down the SC program and generating undifferentiated cells. We observed a similar effect in the developing epithelium and in adult mucosa where enhanced levels of H3K27me3 were observed in crypt SCs upon *Taf4* inactivation, in line with the idea of increased PRC2 activity impairing SC function in vivo.

An additional consequence of *Taf4* loss in the adult mucosa, in *Apc*-driven tumors and, to a lesser extent, in the embryonic intestine is a modified inflammatory/immune microenvironment. These changes reflected altered immune composition that was most prominently seen in the tumors characterized by higher numbers of mast cells/basophils but reduced numbers of T lymphocytes confirmed by the strong reduction in their key markers and by strongly reduced IFNα/γ signaling. High levels of mast cells have been associated with increased tumor growth in human colon cancer [39], whereas a low rate of cytotoxic T cell recruitment correlates with compromised immunosurveillance and poor prognosis in colon cancer [21, 40]. Hence the increased number of tumors seen upon *Taf4* inactivation likely results from reduced immunosurveillance. Several lines of evidence suggest that the increased PRC2 activity seen upon *Taf4* inactivation may be linked to the altered mucosal and tumor immune microenvironment. Previous studies relate increased Ezh2 expression and PRC2 activity to immunoediting of the tumor environment [41, 42]. Specifically, increased PRC2 activity in colon cancer cells reduced T-cell migration to tumors [43] consistent with our observations. IFNγ signaling and antigen presentation is also consistent with the reduced MHC class II seen in the *Taf4*-null tumors [21, 41]. We therefore propose that increased PRC2 activity in the adult intestinal mucosa alters its immune microenvironment providing an environment that promotes *Apc*-driven tumorigenesis through reduced immune surveillance. Changes in the immune microenvironment, in particular the reduction of IFNα signaling which is protective in acute colitis [44], may also be involved in the hypersensitivity of *Taf4*-deficient mice to DSS-induced inflammatory injury.

Together, our observations describe for the first time a novel function of Taf4 that antagonizes PRC2-mediated repression of the SC gene expression program to assure normal development, homeostasis, and immune microenvironment of the intestine epithelium.

## Material and methods

### Mice and treatments

Mice experiments were performed in the certified animal facility (#G/H-67-482-21) according to the protocol approved by the French Ministry of Agriculture under the permit APAFiS #14197. *Taf4*^*lox/lox*^ [25], *VilCre* and *VilCreER*^*T2*^ [45], *Lgr5-GFP-CreER*^*T2*^ [12], and *Apc*^*Δ14/+*^ [46] mice have been described. Animals were genotyped by PCR on tail DNA with the following primers: *Taf4*^*lox/lox*^ allele CTAGTTACTGCTCTGCACAAT/GTGCTCCATGACTCTGGCAAG/CAGCCAAAGCTACATAATAAGT; *VilCre* and *VilCreER*^*T2*^ alleles CAAGCCTGGCTCGACGGCC/CGCGAACATCTTCAGGTTCT; *Lgr5-GFP-CreER*^*T2*^ allele CTGCTCTCTGCTCCCAGTCT/ATACCCCATCCCTTTTGAGC/GAACTTCAGGGTCAGCTTGC; *Apc*^*wt*^ allele CTGTTCTGCAGTATGTTATCA/CTATGAGTCAACACAGGATTA; *Apc*^*Δ14*^ allele CTGTTCTGCAGTATGTTATCA/TATAAGGGCTAACAGTCAATA.

For conditional inactivation of the *Taf4* gene, *Taf4*^*lox/lox*^*::VilCreER*^*T2*^ mice, *Taf4*^*lox/lox*^*::Lgr5-GFP-CreER*^*T2*^ mice or *Taf4*^*lox/lox*^*::VilCreER*^*T2*^*::Apc*^*Δ14/+*^ mice aged 2–3 months received intraperitoneal injections of 1.6 mg Tamoxifen (Sigma-Aldrich) in corn oil, once daily for 3 days. Controls, either wild types or *Taf4*^*lox/lox*^ or *VilCreER*^*T2*^ or *Lgr5-GFP-CreER*^*T2*^ or *Apc*^*Δ14/+*^, also received Tamoxifen.

Animals were euthanized when body weight loss reached 20% of initial body weight.

BrdU pulse-chase labeling experiments were performed on *Taf4*^*lox/lox*^*::VilCreER*^*T2*^ and control *Taf4*^*lox/lox*^ mice treated 10 days earlier with Tamoxifen and injected (day 0) with a single dose of 1 mg BrdU (Sigma-Aldrich). Animals were euthanized at day 1, 2 or 3 after BrdU administration.

Paracellular and transcellular permeability was determined by measuring the serum levels of fluorescein isothiocyanate conjugated dextran (FITC-Dextan 4.4 kDa, Sigma-Aldrich) and D-Xylose (Sigma-Aldrich) after gavage. Briefly, 10 days after Tamoxifen treatment, *Taf4*^*lox/lox*^*::VilCreER*^*T2*^ and control *Taf4*^*lox/lox*^ mice were starved during 4 h and then force-fed with 500 mg/kg FITC-Dextran or 2 g/kg D-Xylose in PBS pH 7.4. Blood samples were taken 3 h after gavage by submandibular collection. The serum levels of FITC-Dextran and D-Xylose were respectively measured by direct spectrophotofluometry and with the D-Xylose Kit (Chrondrex, 6601) coupled with spectrophotometry, using the spectrophometer TriStar Multimode reader LB 942 (BERTHOLD Technologies).

For gut inflammation studies, *Taf4*^*lox/lox*^*::VilCreER*^*T2*^ and control *Taf4*^*lox/lox*^ mice 12 days after the Tamoxifen treatment were given 2% Dextran Sulfate Sodium (DSS 36–50 kDa, MP Biomedicals, Illkirch, France) in drinking water for 7 days and then tap water for 3 days. Throughout the experiment, mice were daily injected with 20 mg/kg Lurocaine (Centravet, Lapalisse, France, LUR003). The presence of blood in the stools was analyzed using HemoCARE (Care Diagnostic, Voerde, Germany). The clinical score was determined daily based on body weight loss, stool consistency and blood in the stools, as described [47]. At the end of the experiment, the colon was removed, flushed with PBS, measured, mounted as Swiss Roll, fixed in 4% paraformaldehyde for 4 h and embedded in paraffin. Assessment of inflammation was performed with regard to stiffness, edema, ulcerations and thickness.

### Enteroid cultures and treatments

Enteroid cultures were established from ileal crypts of 4-month-old *Taf4*^*lox/lox*^*::VilCreER*^*T2*^ and control *Taf4*^*lox/lox*^ mice, not treated with Tamoxifen. Ileal fragments were incubated in Gentle Cell Dissociation Reagent (STEMCELL™, 07174) for 15 min, then ~50 crypts were embedded in 20 µl of Matrigel® (Corning®, #356231) in 48-wells plates (Greiner Bio-one, 677180) and grown in 250 µl Mouse IntestiCult™ Organoid Growth Medium (STEMCELL™, 06005). Enteroids were passed every week after mechanical breakage with a 200 µl pipette and dilution at 1:4 for maintenance and RNA extraction or at 1:8 for immunolabelling and kinetics studies. Experiments were performed on enteroids established for at least 5 passages. For early *Taf4* gene inactivation, enteroid fragments were plated for 2 h and then treated for 3 days with 1 µM (Z)-4-hydroxytamoxifen (4-OHT, Sigma-Aldrich, H7904) in EtOH or with EtOH alone for control experiments. During the 3-days treatment, fresh IntestiCult™ medium with 4-OHT was changed every day and then every 2 days without 4-OHT up the end of the experiment. For late *Taf4* gene inactivation, enteroids were grown for 5 days in IntestiCult™ medium, then treated for 3 days with 4-OHT in EtOH or EtOH alone as described above, and then cultured in IntestiCult™ medium without 4-OHT up the end of the experiment. When indicated, EPZ6438 at 1 µM (MedChemExpress, HY-13803) in DMSO was added to the culture medium at the same times as 4-OHT and then changed with fresh IntestiCult™ medium up to the end of the experiment.

### Immunostaining of tissue samples and enteroids

Antibodies used for this study are listed in Supplementary Table 12.

Intestinal samples taken from mouse embryos and adult animals were fixed in 4% paraformaldehyde and embedded in paraffin, and then analyzed by immunohistochemistry and immunofluorescence detection as previously described [48]. To determine the proportion of labeled cells, at least three pictures were taken per animal in five animals and cell counting was performed on a minimum of 1000 cells per picture. Pictures were taken either with an Axiophot microscope or an Axio Imager Z2 microscope (Zeiss).

Enteroids grown in IntestiCult^TM^ medium and Matrigel in 8-wells Lab-Tek® Chamber Slide™ (Dominique Dutscher) were directly fixed for 30 min with 4% paraformaldehyde, 60 mM PIPES, 25 mM HEPES, 10 mM EGTA, 2 mM magnesium acetate and permeabilized for 30 min in 0.5% Triton X-100 (Euromedex). After blockade for 2 h at 37 °C in 5% BSA (Euromedex), primary antibodies were added and incubated 2 h at room temperature followed by overnight at 4 °C. Secondary antibodies were incubated for 2 h at 37 °C. Nuclei were stained with 40,6-diamidino-2-phenylindole dihydrochloride (DAPI) and actin was revealed by Phalloidin-TRITC (Sigma-Aldrich, P1951). Samples were mounted in ProLong™ Gold Antifade Mountant (Life Technologie™, P36930) and analyzed with an Axio Zoom.V16 microscope (Zeiss) for stereomicroscopy or with an Axio Imager M2 microscope coupled to a Hamamatsu’s camera Orca Flash 4v3 using the ApoTome.2 function (Zeiss) for optical sectioning.

### Bacterial 16S RNA analysis

The luminal content was collected in the cecum of 8 5-month-old *Taf4*^*lox/lox*^*::VilCreER*^*T2*^ and 8 control *Taf4*^*lox/lox*^ mice treated with Tam at the age of 3 months. DNA extraction was performed using NucleoSpin® DNA Stool kit (Macherey-Nagel—740472.5) adapted with a mechanical lysis step (Fastprep—6.5 m s^−1^ for 2 min). The V3–V4 region of the 16S rRNA gene was amplified with the primers TACGGRAGGCAGCAG/ATCTTACCAGGGTATCTAATCCT according to GeT-PlaGe platform protocol (INRAE). Sequencing was performed on Illumina MiSeq system using 2*300 bp paired-end mode. For sequence data analysis, remaining adapter/primer sequences were trimmed and reads were checked for quality (≥20) and length (≥200 bp) using Cutadapt [49]. Reads were further corrected for known sequencing errors using SPAdes [50] and then merged using PEAR [51]. OTU were identified using the Vsearch pipeline [52] set up to dereplicate, cluster, chimera check the merged reads. OTU taxonomical classification was performed using classifier from the RDPTools suit [53]. Statistical tests were performed using Wilcoxon Rank Sum Test for group comparison. Multiple tests were corrected using the False Discovery Rate method (*q* value) as required.

### Bulk RNA preparation and RNA-seq

RNA was extracted from the small intestine of 3 E17.5 *Taf4*^*lox/lox*^*::VilCre* and 3 control *Taf4*^*lox/lox*^ littermates and from 3 adult *Taf4*^*lox/lox*^*::VilCreER*^*T2*^ and 3 control *Taf4*^*lox/lox*^ mice 10 days after Tamoxifen administration. RNA was also extracted from 3 wells of 4-OHT-treated *Taf4*^*lox/lox*^*::VilCreER*^*T2*^ and control *Taf4*^*lox/lox*^ enteroids at day 3 of culture, and from 3 wells of *Taf4*^*lox/lox*^*::VilCreER*^*T2*^ enteroids at day 15 of culture after treatment with 4-OHT and EPZ6438. RNA preparation used Tri Reagent (Euromedex) and the quality was analyzed using nanoRNA chips on a Bioanalyser 2100 (Agilent Technologies). Complementary DNA was generated and linearly amplified from 3 ng total RNA using the Ovation RNA-seq V2 system (NuGEN technologies Inc., Leek, The Netherlands), according to the manufacturer’s instructions. The amplified cDNA was then purified using Agencourt AMPure XP beads (Beckman-Coulter, Villepinte, France) in a 1.8:1 bead to sample ratio and fragmented by sonication using a Covaris E220 instrument (with duty cycle: 10%, maximum incident power: 175 watts and cycles/burst: 200 for 120 s). The RNA-seq libraries were generated from 100 ng fragmented cDNA using the Ovation Ultralow v2 library system (NuGEN technologies Inc., Leek, The Netherlands) according to the manufacturer’s instructions, with 6 PCR cycles for library amplification. The final libraries were verified for quality and quantified using capillary electrophoresis before sequencing on an Illumina Hi-Seq4000.

Reads were preprocessed to remove adapter and low-quality sequences (Phred quality score below 20). After this preprocessing, reads shorter than 40 bases were discarded from further analysis. These preprocessing steps were performed using Cutadapt version 1.10 [49]. Reads were mapped to rRNA sequences using Bowtie version 2.2.8 [54], and reads mapping to rRNA sequences were removed for further analysis. Reads were mapped onto the mm9 assembly of Mus musculus genome using STAR version 2.5.3a [55]. Gene expression quantification was performed from uniquely aligned reads using Htseq-count version 0.6.1p1 [56], with annotations from Ensembl version 67 and “union” mode. Only non-ambiguously assigned reads have been retained for further analyses. Read counts have been normalized across samples with the median-of-ratios method [57], to make these counts comparable between samples. Comparisons of interest were performed using the Wald test for differential expression and implemented in the Bioconductor package DESeq2 version 1.16.1 [58]. Genes with high Cook’s distance were filtered out and independent filtering based on the mean of normalized counts was performed. *p* values were adjusted for multiple testing using the Benjamini and Hochberg method [59]. Heatmaps were generated with R-package pheatmap v1.0.12. Deregulated genes were defined as genes with log_2_(FoldChange) >1 or <−1 and adjusted *p* value <0.05.

### Assay for transposase-accessible chromatin (ATAC-seq)

ATAC-seq was performed at day 3 of culture from 20,000 cells of *Taf4*^*lox/lox*^*::VilCreER*^*T2*^ and control *Taf4*^*lox/lox*^ enteroids treated with 4-OHT. Sequenced reads were mapped to the mouse genome assembly mm9 using Bowtie [54] with the following arguments: “-m 1 -strata -best -y -S -l 40 -p 2”.

After sequencing, peak detection was performed using the MACS software [60] v2.1.1.20160309 with arguments “-nomodel -shift −100 -extsize 200 -broad”. Peaks were annotated with Homer [61] using the GTF from ENSEMBL v67. Peak intersections were computed using Bedtools [62]. Global Clustering was done using seqMINER [63]. De novo motif discovery was performed using the MEME suite [64]. Footprinting signatures were calculated using TOBIAS v0.5.1 [15], and differential footprinting scores were plotted with R-package ggplot2 [65].

### Single-cell RNA-seq (scRNA-seq)

*Taf4*^*lox/lox*^*::VilCreER*^*T2*^ and control *Taf4*^*lox/lox*^ enteroids treated with 4-OHT from day 5 to 8 of culture were dissociated at the end of the 4-OHT treatment with Accutase (A6964, Sigma) at 27 °C for 5 min and the cells were suspended in culture medium. Cells were then sorted by flow cytometry to select live cells and captured using 10X Genomics Chromium Analyzer. After sequencing, raw reads were processed using CellRanger (v 3.1) to align on the mm10 mouse genome, remove unexpressed genes and quantify barcodes and UMIs. Data were then analyzed in R (v3.6.3) using Seurat v3.1.4 [66]. First cells were filtered, only cells with feature count ranging from 200 to 6000 and with percentage of mitochondrial reads <15% were kept for the analysis. Then counts were normalized with the “LogNormalize” method and scaled to remove unwanted sources of variation. Clustering was performed on variable features using the 20 most significant principal components and a resolution of 0.9. Analysis of regulome was performed using SCENIC v1.1.2.2 [67].

## Supplementary information

Supp Figs Legends

Supp Fig 1

Supp Fig 2

Supp Fig 3

Supp Fig 4

Supp Fig 5

Supp Fig 6

Supp Table 1

Supp Table 2

Supp Table 3

Supp Table 4

Supp Table 5

Supp Table 6

Supp Table 7

Supp Table 8

Supp Table 9

Supp Table 10

Supp Table 11

Supp Table 12

checklist

## Data Availability

16S rRNA data are publicly available from NCBI SRA under the Bioproject accession number PRJNA842218. RNA-seq, sc-RNA-seq and ATAC-seq data are deposited in the GEO database under the accession number GSE205442.

## References

[1] Gehart H, Clevers H (2019). Tales from the crypt: new insights into intestinal stem cells. Nat Rev Gastroenterol Hepatol.

[2] Langer D, Martianov I, Alpern D, Rhinn M, Keime C, Dollé P (2016). Essential role of the TFIID subunit TAF4 in murine embryogenesis and embryonic stem cell differentiation. Nat Commun.

[3] Fadloun A, Kobi D, Pointud J-C, Indra AK, Teletin M, Bole-Feysot C (2007). The TFIID subunit TAF4 regulates keratinocyte proliferation and has cell-autonomous and non-cell-autonomous tumour suppressor activity in mouse epidermis. Development.

[4] Alpern D, Langer D, Ballester B, Le Gras S, Romier C, Mengus G (2014). TAF4, a subunit of transcription factor II D, directs promoter occupancy of nuclear receptor HNF4A during post-natal hepatocyte differentiation. Elife.

[5] Kleiber T, Davidson G, Mengus G, Martianov I, Davidson I (2021). Single cell transcriptomics reveal trans-differentiation of pancreatic beta cells following inactivation of the TFIID subunit Taf4. Cell Death Dis.

[6] Muñoz J, Stange DE, Schepers AG, van de Wetering M, Koo B-K, Itzkovitz S (2012). The Lgr5 intestinal stem cell signature: robust expression of proposed quiescent ‘+4’ cell markers. EMBO J.

[7] Petitprez F, Levy S, Sun C-M, Meylan M, Linhard C, Becht E (2020). The murine microenvironment cell population counter method to estimate abundance of tissue-infiltrating immune and stromal cell populations in murine samples using gene expression. Genome Med.

[8] Wu M, Li P, Li J, An Y, Wang M, Zhong G (2020). The differences between luminal microbiota and mucosal microbiota in mice. J Microbiol Biotechnol.

[9] Mosca A, Leclerc M, Hugot JP. Gut microbiota diversity and human diseases: should we reintroduce key predators in our ecosystem? Front Microbiol. 2016;7. 10.3389/fmicb.2016.00455. 10.3389/fmicb.2016.00455 PMC4815357 27065999

[10] Sprangers J, Zaalberg IC, Maurice MM (2021). Organoid-based modeling of intestinal development, regeneration, and repair. Cell Death Differ.

[11] Rona G, Roberti D, Yin Y, Pagan JK, Homer H, Sassani E (2018). PARP1-dependent recruitment of the FBXL10-RNF68-RNF2 ubiquitin ligase to sites of DNA damage controls H2A.Z loading. Elife.

[12] Barker N, van Es JH, Kuipers J, Kujala P, van den Born M, Cozijnsen M (2007). Identification of stem cells in small intestine and colon by marker gene Lgr5. Nature.

[13] Sato T, Vries RG, Snippert HJ, van de Wetering M, Barker N, Stange DE (2009). Single Lgr5 stem cells build crypt-villus structures in vitro without a mesenchymal niche. Nature.

[14] Schuijers J, van der Flier LG, van Es J, Clevers H (2014). Robust cre-mediated recombination in small intestinal stem cells utilizing the olfm4 locus. Stem Cell Rep.

[15] Bentsen M, Goymann P, Schultheis H, Klee K, Petrova A, Wiegandt R (2020). ATAC-seq footprinting unravels kinetics of transcription factor binding during zygotic genome activation. Nat Commun.

[16] Asfaha S, Hayakawa Y, Muley A, Stokes S, Graham TA, Ericksen RE (2015). Krt19(+)/Lgr5(-) cells are radioresistant cancer-initiating stem cells in the colon and intestine. Cell Stem Cell.

[17] Ayyaz A, Kumar S, Sangiorgi B, Ghoshal B, Gosio J, Ouladan S (2019). Single-cell transcriptomes of the regenerating intestine reveal a revival stem cell. Nature.

[18] Montgomery RK, Carlone DL, Richmond CA, Farilla L, Kranendonk MEG, Henderson DE (2011). Mouse telomerase reverse transcriptase (mTert) expression marks slowly cycling intestinal stem cells. Proc Natl Acad Sci USA.

[19] Gregorieff A, Liu Y, Inanlou MR, Khomchuk Y, Wrana JL (2015). Yap-dependent reprogramming of Lgr5(+) stem cells drives intestinal regeneration and cancer. Nature.

[20] Murata K, Jadhav U, Madha S, van Es J, Dean J, Cavazza A (2020). Ascl2-dependent cell dedifferentiation drives regeneration of ablated intestinal stem cells. Cell Stem Cell.

[21] Du W, Frankel TL, Green M, Zou W (2022). IFNγ signaling integrity in colorectal cancer immunity and immunotherapy. Cell Mol Immunol.

[22] Gao Q, Wang S, Chen X, Cheng S, Zhang Z, Li F (2019). Cancer-cell-secreted CXCL11 promoted CD8+ T cells infiltration through docetaxel-induced-release of HMGB1 in NSCLC. J Immunother Cancer.

[23] Chen J, Ye X, Pitmon E, Lu M, Wan J, Jellison ER (2019). IL-17 inhibits CXCL9/10-mediated recruitment of CD8+ cytotoxic T cells and regulatory T cells to colorectal tumors. J Immunother Cancer.

[24] Wang K, Kim MK, Di Caro G, Wong J, Shalapour S, Wan J (2014). Interleukin-17 receptor a signaling in transformed enterocytes promotes early colorectal tumorigenesis. Immunity.

[25] Mengus G, Fadloun A, Kobi D, Thibault C, Perletti L, Michel I (2005). TAF4 inactivation in embryonic fibroblasts activates TGF beta signalling and autocrine growth. EMBO J.

[26] Yu Y, Chen S, Lu G-F, Wu Y, Mo L, Liu Z-Q (2014). Alphavbeta6 is required in maintaining the intestinal epithelial barrier function. Cell Biol Int.

[27] Hageman JH, Heinz MC, Kretzschmar K, van der Vaart J, Clevers H, Snippert HJG (2020). Intestinal regeneration: regulation by the microenvironment. Dev Cell.

[28] Farin HF, Jordens I, Mosa MH, Basak O, Korving J, Tauriello DVF (2016). Visualization of a short-range Wnt gradient in the intestinal stem-cell niche. Nature.

[29] Erkek S, Johann PD, Finetti MA, Drosos Y, Chou H-C, Zapatka M (2019). Comprehensive analysis of chromatin states in atypical teratoid/rhabdoid tumor identifies diverging roles for SWI/SNF and Polycomb in gene regulation. Cancer Cell.

[30] Nakayama RT, Pulice JL, Valencia AM, McBride MJ, McKenzie ZM, Gillespie MA (2017). SMARCB1 is required for widespread BAF complex-mediated activation of enhancers and bivalent promoters. Nat Genet.

[31] Valencia AM, Kadoch C (2019). Chromatin regulatory mechanisms and therapeutic opportunities in cancer. Nat Cell Biol.

[32] Lorzadeh A, Romero-Wolf M, Goel A, Jadhav U (2021). Epigenetic regulation of intestinal stem cells and disease: a balancing act of DNA and histone methylation. Gastroenterology.

[33] Piunti A, Shilatifard A (2021). The roles of Polycomb repressive complexes in mammalian development and cancer. Nat Rev Mol Cell Biol.

[34] Chiacchiera F, Rossi A, Jammula S, Piunti A, Scelfo A, Ordóñez-Morán P (2016). Polycomb complex PRC1 preserves intestinal stem cell identity by sustaining Wnt/β-catenin transcriptional activity. Cell Stem Cell.

[35] Chiacchiera F, Rossi A, Jammula S, Zanotti M, Pasini D (2016). PRC2 preserves intestinal progenitors and restricts secretory lineage commitment. EMBO J.

[36] Koppens MAJ, Bounova G, Gargiulo G, Tanger E, Janssen H, Cornelissen-Steijger P, et al. Deletion of Polycomb repressive complex 2 from mouse intestine causes loss of stem cells. Gastroenterology. 2016. 10.1053/j.gastro.2016.06.020. 10.1053/j.gastro.2016.06.020 27342214

[37] Serra D, Mayr U, Boni A, Lukonin I, Rempfler M, Challet Meylan L (2019). Self-organization and symmetry breaking in intestinal organoid development. Nature.

[38] Nakanishi Y, Reina-Campos M, Nakanishi N, Llado V, Elmen L, Peterson S (2016). Control of paneth cell fate, intestinal inflammation, and tumorigenesis by PKCλ/ι. Cell Rep.

[39] Yu Y, Blokhuis B, Derks Y, Kumari S, Garssen J, Redegeld F (2018). Human mast cells promote colon cancer growth via bidirectional crosstalk: studies in 2D and 3D coculture models. Oncoimmunology.

[40] Galon J, Costes A, Sanchez-Cabo F, Kirilovsky A, Mlecnik B, Lagorce-Pages C (2006). Type, density, and location of immune cells within human colorectal tumors predict clinical outcome. Science.

[41] Sun S, Yu F, Xu D, Zheng H, Li M (2022). EZH2, a prominent orchestrator of genetic and epigenetic regulation of solid tumor microenvironment and immunotherapy. Biochim Biophys Acta Rev Cancer.

[42] Kang N, Eccleston M, Clermont P-L, Latarani M, Male DK, Wang Y (2020). EZH2 inhibition: a promising strategy to prevent cancer immune editing. Epigenomics.

[43] Nagarsheth N, Peng D, Kryczek I, Wu K, Li W, Zhao E (2016). PRC2 epigenetically silences Th1-type chemokines to suppress effector T-cell trafficking in colon cancer. Cancer Res.

[44] Li J-Y, Xiao J, Gao M, Zhou H-F, Fan H, Sun F (2021). IRF/Type I IFN signaling serves as a valuable therapeutic target in the pathogenesis of inflammatory bowel disease. Int Immunopharmacol.

[45] El Marjou F, Janssen KP, Chang BH, Li M, Hindie V, Chan L (2004). Tissue-specific and inducible Cre-mediated recombination in the gut epithelium. Genesis.

[46] Colnot S, Niwa-Kawakita M, Hamard G, Godard C, Le Plenier S, Houbron C (2004). Colorectal cancers in a new mouse model of familial adenomatous polyposis: influence of genetic and environmental modifiers. Lab Invest.

[47] Li Y, Zhang M, Xiao H, Fu H, Ho A, Lin C (2015). Addition of berberine to 5-aminosalicylic acid for treatment of dextran sulfate sodium-induced chronic colitis in C57BL/6 mice. PLoS ONE.

[48] Balbinot C, Armant O, Elarouci N, Marisa L, Martin E, De Clara E (2018). The Cdx2 homeobox gene suppresses intestinal tumorigenesis through non-cell-autonomous mechanisms. J Exp Med.

[49] Martin M (2011). Cutadapt removes adapter sequences from high-throughput sequencing reads. EMBnet J.

[50] Bankevich A, Nurk S, Antipov D, Gurevich AA, Dvorkin M, Kulikov AS (2012). SPAdes: a new genome assembly algorithm and its applications to single-cell sequencing. J Comput Biol.

[51] Zhang J, Kobert K, Flouri T, Stamatakis A (2014). PEAR: a fast and accurate Illumina Paired-End reAd mergeR. Bioinformatics.

[52] Rognes T, Flouri T, Nichols B, Quince C, Mahé F (2016). VSEARCH: a versatile open source tool for metagenomics. PeerJ.

[53] Cole JR, Wang Q, Cardenas E, Fish J, Chai B, Farris RJ (2009). The Ribosomal Database Project: improved alignments and new tools for rRNA analysis. Nucleic Acids Res.

[54] Langmead B, Trapnell C, Pop M, Salzberg SL (2009). Ultrafast and memory-efficient alignment of short DNA sequences to the human genome. Genome Biol.

[55] Dobin A, Davis CA, Schlesinger F, Drenkow J, Zaleski C, Jha S (2013). STAR: ultrafast universal RNA-seq aligner. Bioinformatics.

[56] Anders S, Pyl PT, Huber W (2015). HTSeq-a Python framework to work with high-throughput sequencing data. Bioinformatics.

[57] Anders S, Huber W (2010). Differential expression analysis for sequence count data. Genome Biol.

[58] Love MI, Huber W, Anders S (2014). Moderated estimation of fold change and dispersion for RNA-seq data with DESeq2. Genome Biol.

[59] Benjamini Y, Hochberg Y (1994). Controlling the false discovery rate: a pratical and powerful ap- proach to multiple testing. Genome Biol.

[60] Zhang Y, Liu T, Meyer CA, Eeckhoute J, Johnson DS, Bernstein BE (2008). Model-based analysis of ChIP-Seq (MACS). Genome Biol.

[61] Heinz S, Benner C, Spann N, Bertolino E, Lin YC, Laslo P (2010). Simple combinations of lineage-determining transcription factors prime cis-regulatory elements required for macrophage and B cell identities. Mol Cell.

[62] Quinlan AR, Hall IM (2010). BEDTools: a flexible suite of utilities for comparing genomic features. Bioinformatics.

[63] Ye T, Krebs AR, Choukrallah M-A, Keime C, Plewniak F, Davidson I (2011). seqMINER: an integrated ChIP-seq data interpretation platform. Nucleic Acids Res.

[64] Bailey TL, Johnson J, Grant CE, Noble WS (2015). The MEME suite. Nucleic Acids Res.

[65] Wickham H. ggplot2: Elegant Graphics for Data Analysis. 2nd ed. Cham: Springer International Publishing: Imprint; 2016. 10.1007/978-3-319-24277-4.

[66] Stuart T, Butler A, Hoffman P, Hafemeister C, Papalexi E, Mauck WM (2019). Comprehensive integration of single-cell data. Cell.

[67] Van de Sande B, Flerin C, Davie K, De Waegeneer M, Hulselmans G, Aibar S (2020). A scalable SCENIC workflow for single-cell gene regulatory network analysis. Nat Protoc.

