# Supplementary material for: General transcription factor TAF4 antagonizes epigenetic silencing by Polycomb to maintain intestine stem cell functions
Source: Cell Death Differ. 2023 Jan 13;30(3):839–53. doi: 10.1038/s41418-022-01109-6 (PMC9984434; doi:10.1038/s41418-022-01109-6)
Supplement: Supplementary file 1 — Supp Figs Legends [file 41418_2022_1109_MOESM1_ESM.docx]

**Legends of supplementary Figures**

**Supplementary Figure 1***.* SCs and *Taf4* inactivation

**A**. Immunofluorescence detection of Olfm4 and Ephb2 in *Taf4^IEC^* and control mice. Bars are 100 µm. **B**. Taf4 protein immunofluorescence detection in *Taf4^CBC^* mice 6 days after Tamoxifen administration. Regular and dotted lines respectively encircle a *Taf4*-not-inactivated and a *Taf4*-inactivated crypt. In the latter, Paneth cells with a half-life of several dozens of days still express the Taf4 protein (white arrowheads). **C**. Absorptive cell differentiation in *Taf4^CBC^* mice. Nuclei are in blue, Taf4 immunostaining in red, and Alkaline Phosphatase activity (Alpi) in green. White arrowheads and open arrowheads respectively show Taf4-depleted and Taf4-expressing nuclei; white arrows and open arrows respectively point to Alpi absent and present at the apical pole of enterocytes. Bars are 50 µm.

**Supplementary Figure 2***.* Effect of *Taf4* inactivation in the adult gut epithelium

**A**. Activated-Capsase3 immunostaining and cell counts in *Taf4^IEC^* and *Taf4^lox/lox^* control (Ctrl) mice 10 days after Tamoxifen injection. Boxes extend from the 25^th^-75^th^ percentile and whiskers represent mean to max. Bars are 100 µm. ns, not significant. **B**. Immunodetection of Cdx2, of the isoforms 1-6 and 7-9 of Hnf4α, and of Hes1 proteins in the intestine of adult *Taf4^IEC^* and *Taf4^lox/lox^* (ctrl) mice 10 days after Tamoxifen administration. Bars: 100 µm.

**Supplementary Figure 3***.* Pathophysiological consequences of *Taf4* inactivation.

**A**. Microbiota. Number of clusters of similar sequence variants of the 16S rDNA gene expressed as OUT (Operational taxonomic units) (left); relative abundance of microbiota families (middle); and genera abundance (right) in the cecum of *Taf4^IEC^* and *Taf4^lox/lox^* control (n=8 for each genotype) mice treated with Tamoxifen 1 month before sample collection. *** *q*<0.001. **B**. Epithelial barrier activity. Blood concentration of FITC-Dextran in *Taf4^IEC^* (n=14) and *Taf4^lox/lox^* control (n=13) mice 3 hours after gavage; ** *p*<0.01. **C**. Sensitivity to acute inflammation induced by 2% DSS in the drinking water. Clinical score of *Taf4^IEC^* (n=5) and *Taf4^lox/lox^* control mice (n=5) during DSS treatment (days 1 to 5) and in the next days (days 6-8). **D**. Histology (HE) and immunohistochemical detection of the indicated proteins in the distal colon of *Taf4^IEC^* and *Taf4^lox/lox^* control mice at the end of the DSS experiment. Bars are 400 µm for histology and 100 µm for immunostaining. In the inflammatory zones, the clot is directly facing the lumen (open arrowhead) or irregularly covered by an injured single-layer epithelium with atrophied glands (black arrowheads).

**Supplementary Figure 4**. Chromatin accessibility changes upon Taf4 loss

**A**. Scatter plot of ATAC-seq peaks in 4-OHT treated *Taf4^IEC^* *vs* *Taf4^lox/lox^* enteroids after early *Taf4* gene inactivation. Blue and red dots respectively represent lower (lost) and higher (gained) peaks in *Taf4^IEC^* *vs* *Taf4^lox/lox^* enteroids. **B**. Read density heat maps of ATAC-seq peaks identifying peaks that were diminished (C7 left panel) or gained (C8 left panel) after Taf4 loss. Center and right panels show re-clustering of C7 and C8, respectively. **C.** In silico footprinting of differentially accessible ATAC-seq sites. **D**. Most frequent DNA binding motifs present in the lost and gained peaks of C7 and C8 identified in 6B. **E**. Ontology of the genes downregulated by Taf4 loss and associated with differentially accessible ATAC-seq peaks.

**Supplementary Figure 5***.* Kinetics of morphological defects in *Taf4^IEC^* enteroids by *Taf4* inactivation and rescue by EPZ6438.

Pictures show the evolution of the same enteroid in each condition from days 1 to 15 of culture. Treatments started at day 1 (**A**) or at day 5 (**B**) of culture. Bars are 200 µm.

**Supplementary Figure 6***.* H3K27me3 changes by *Taf4* inactivation in mouse embryos and adults.

Immunodetection of H3K27me3 in E18.5 *Taf4^IEndoC^* and *Taf4^lox/lox^* control (Ctrl) embryos (**A**) and in adult *Taf4^IEC^* and *Taf4^lox/lox^* control (Ctrl) mice (**B**). Bars are 50 µm.
