# Supplementary figures and images for: General transcription factor TAF4 antagonizes epigenetic silencing by Polycomb to maintain intestine stem cell functions
Source: Cell Death Differ. 2023 Jan 13;30(3):839–53. doi: 10.1038/s41418-022-01109-6 (PMC9984434; doi:10.1038/s41418-022-01109-6)

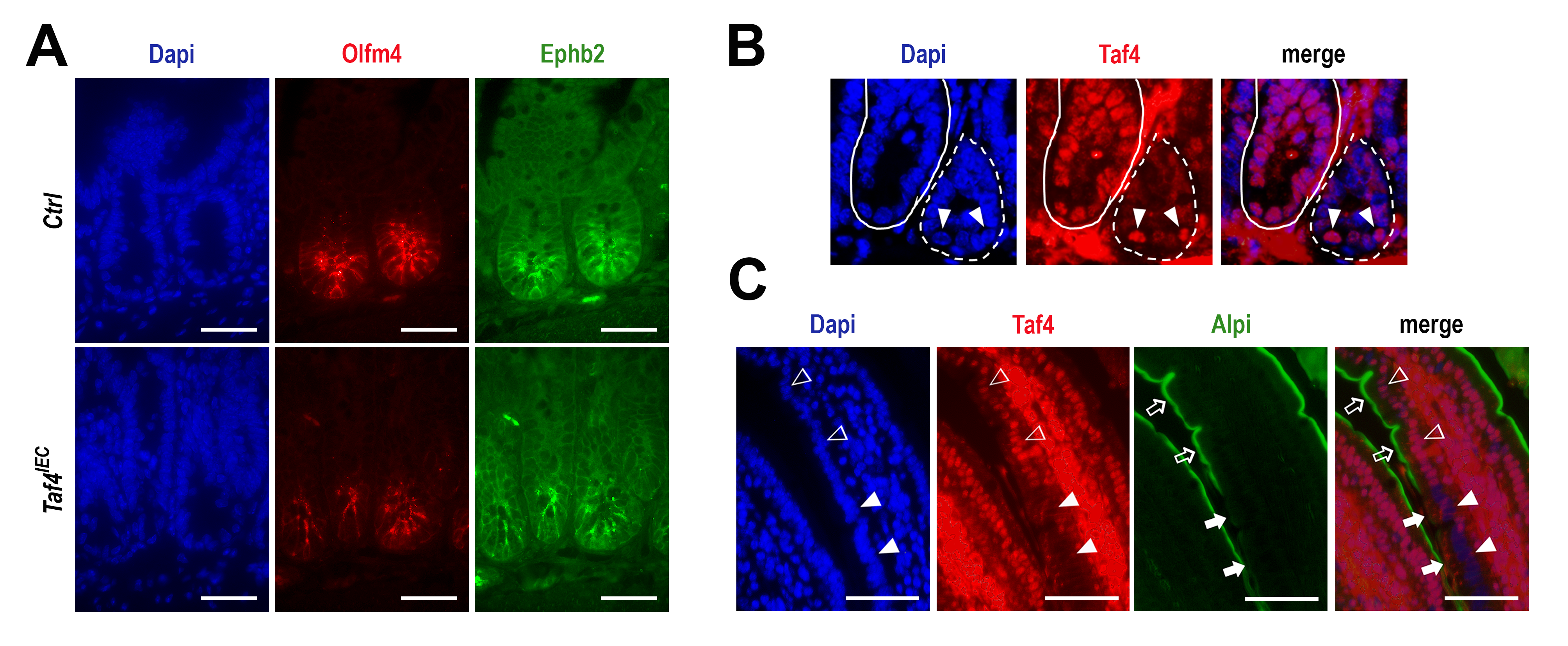

Supplement: Supplementary file 2 — Supp Fig 1 [file 41418_2022_1109_MOESM2_ESM.tif]

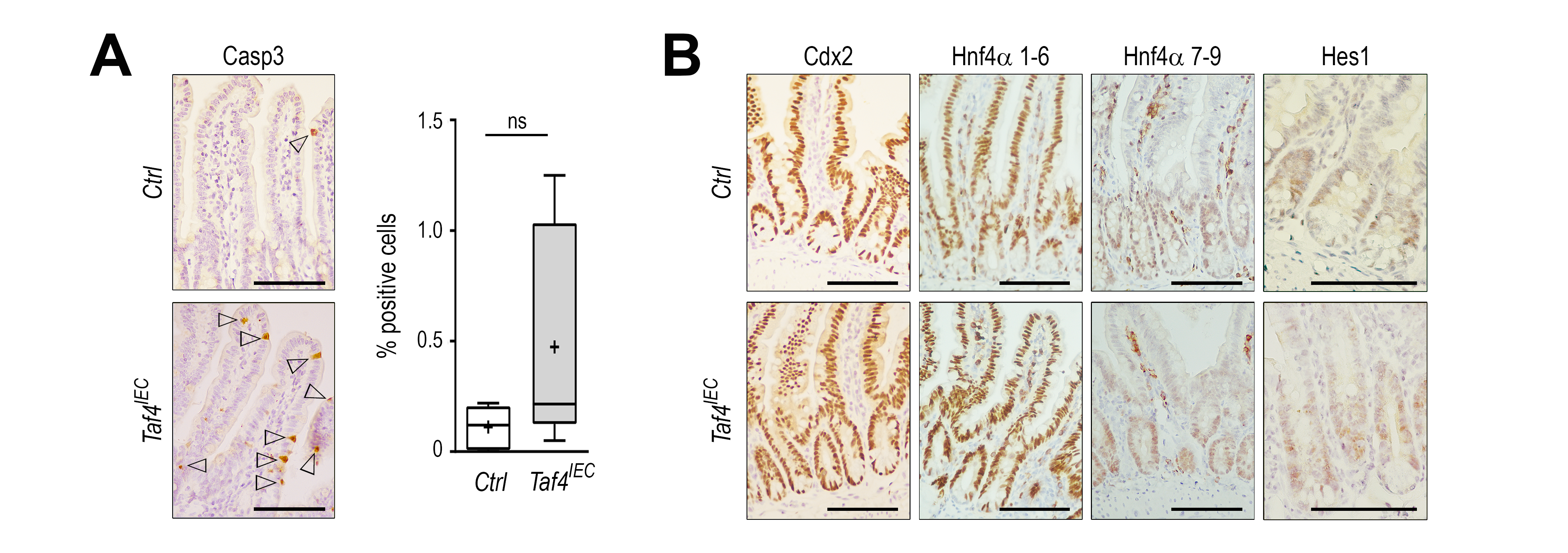

Supplement: Supplementary file 3 — Supp Fig 2 [file 41418_2022_1109_MOESM3_ESM.tif]

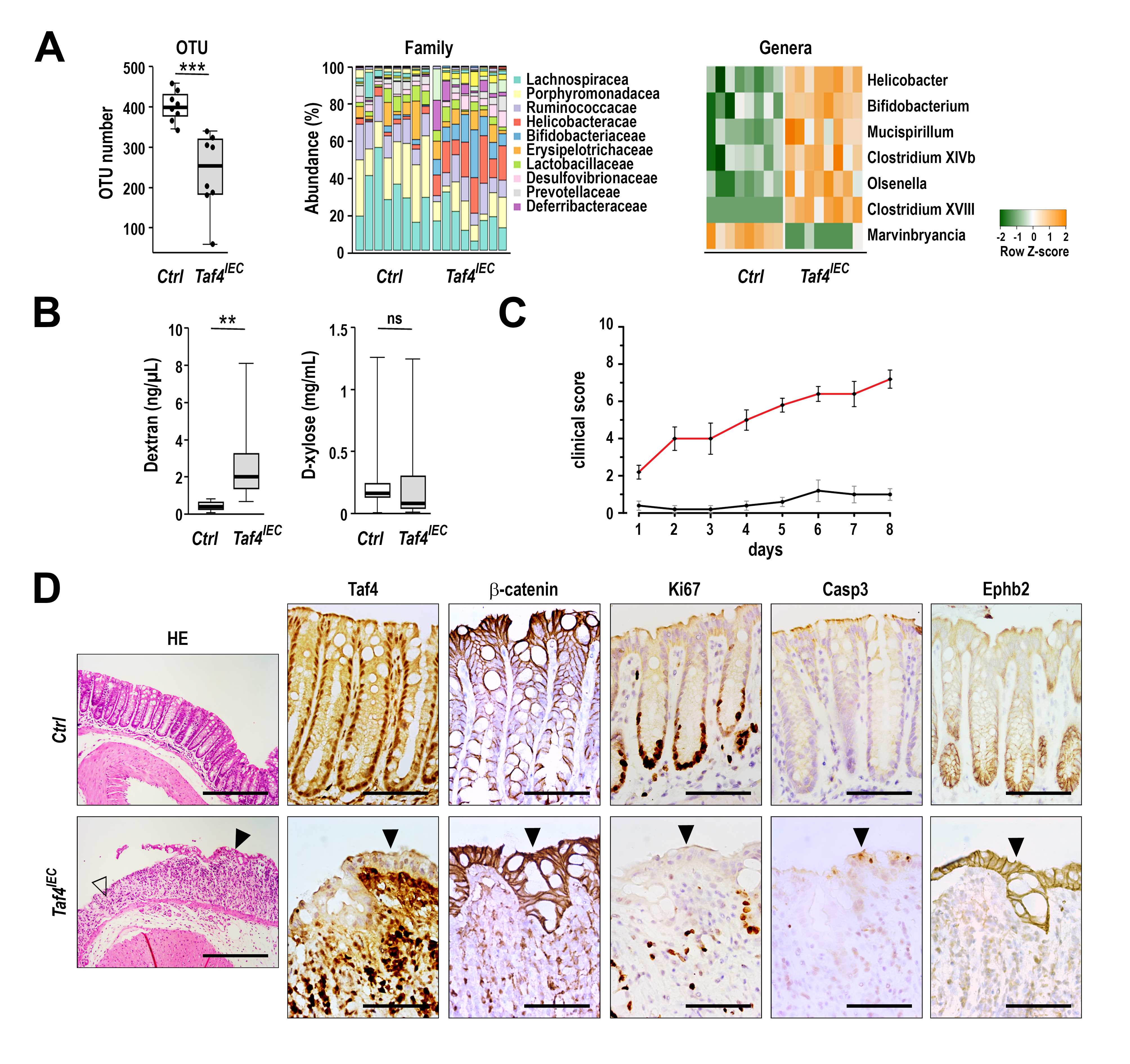

Supplement: Supplementary file 4 — Supp Fig 3 [file 41418_2022_1109_MOESM4_ESM.tif]

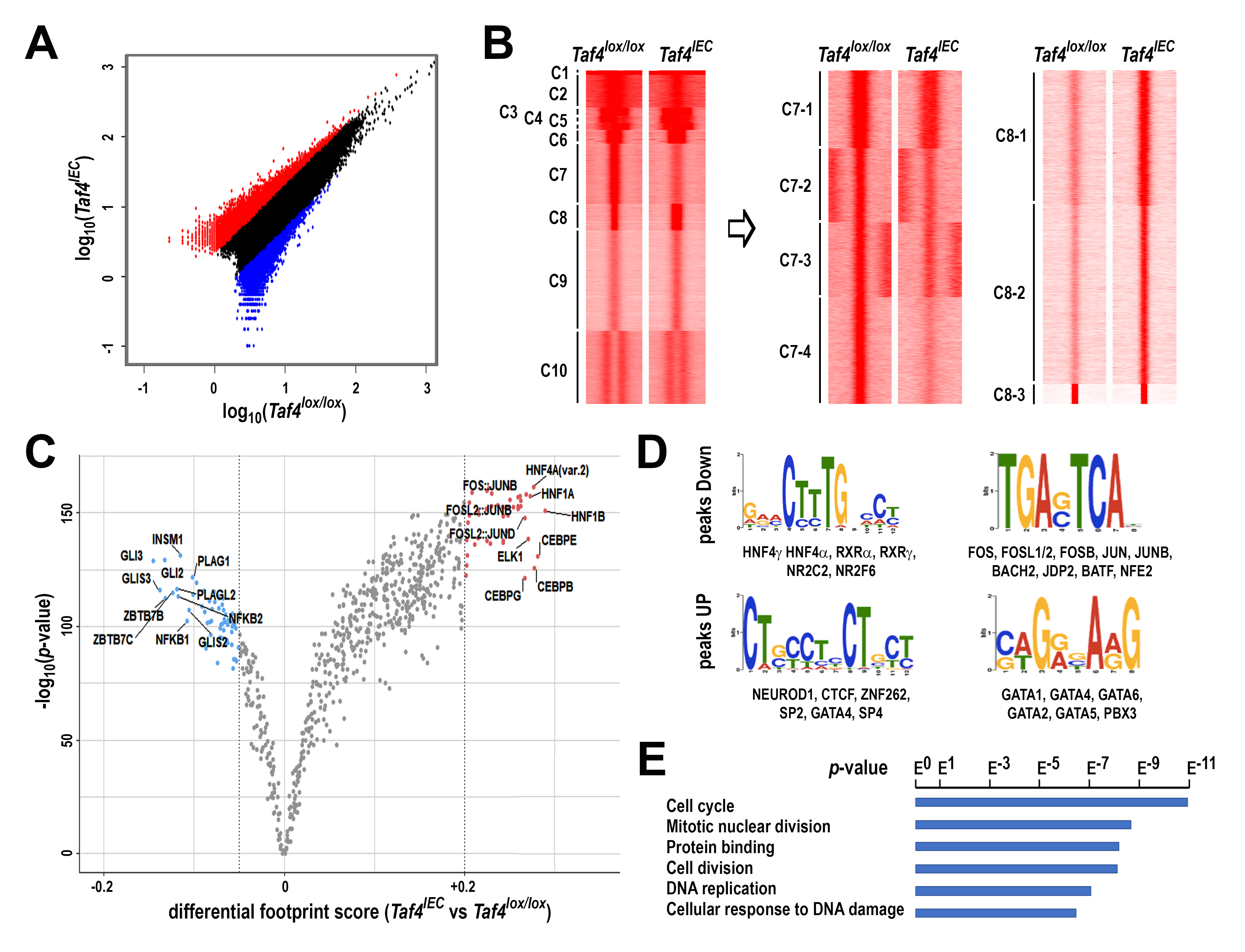

Supplement: Supplementary file 5 — Supp Fig 4 [file 41418_2022_1109_MOESM5_ESM.tif]

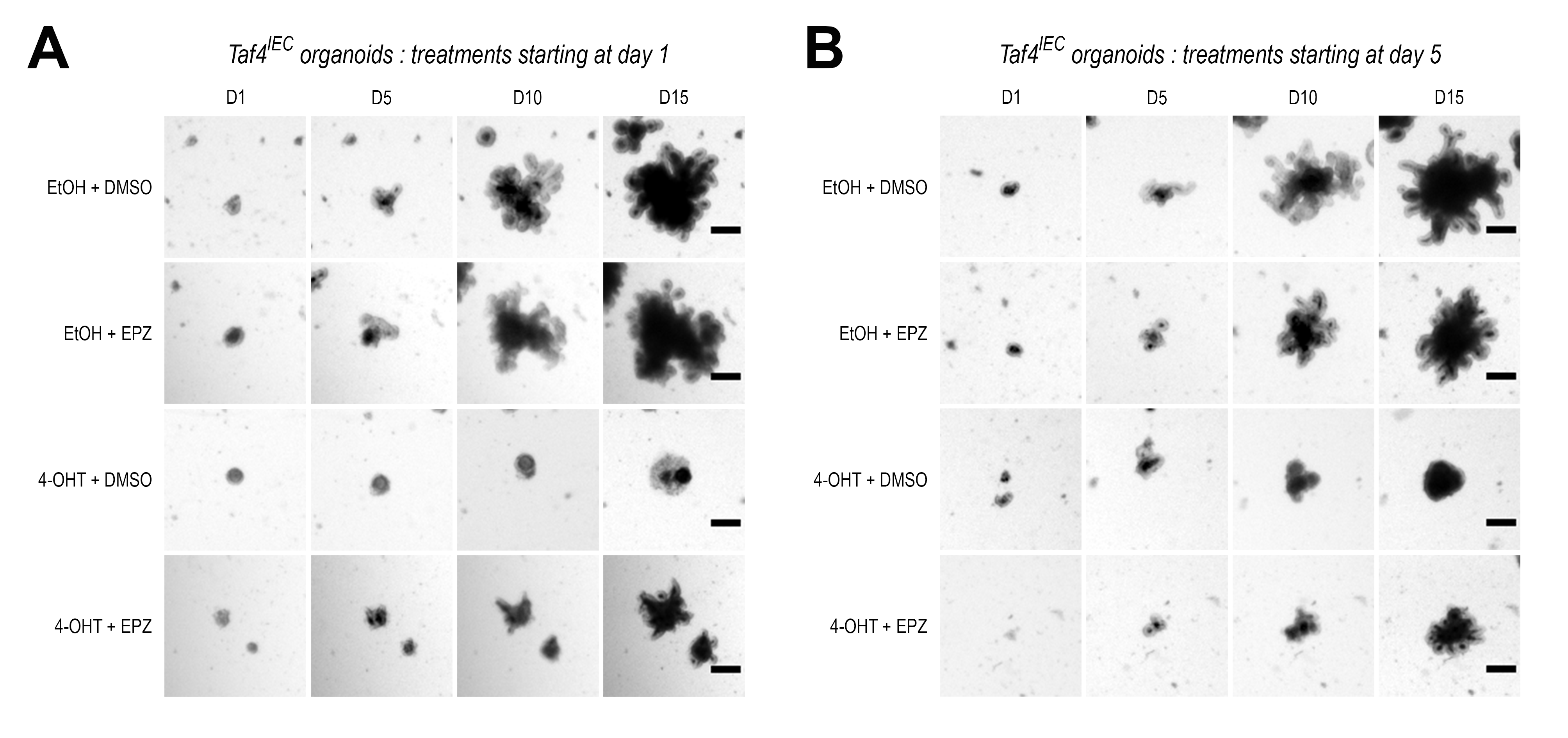

Supplement: Supplementary file 6 — Supp Fig 5 [file 41418_2022_1109_MOESM6_ESM.tif]

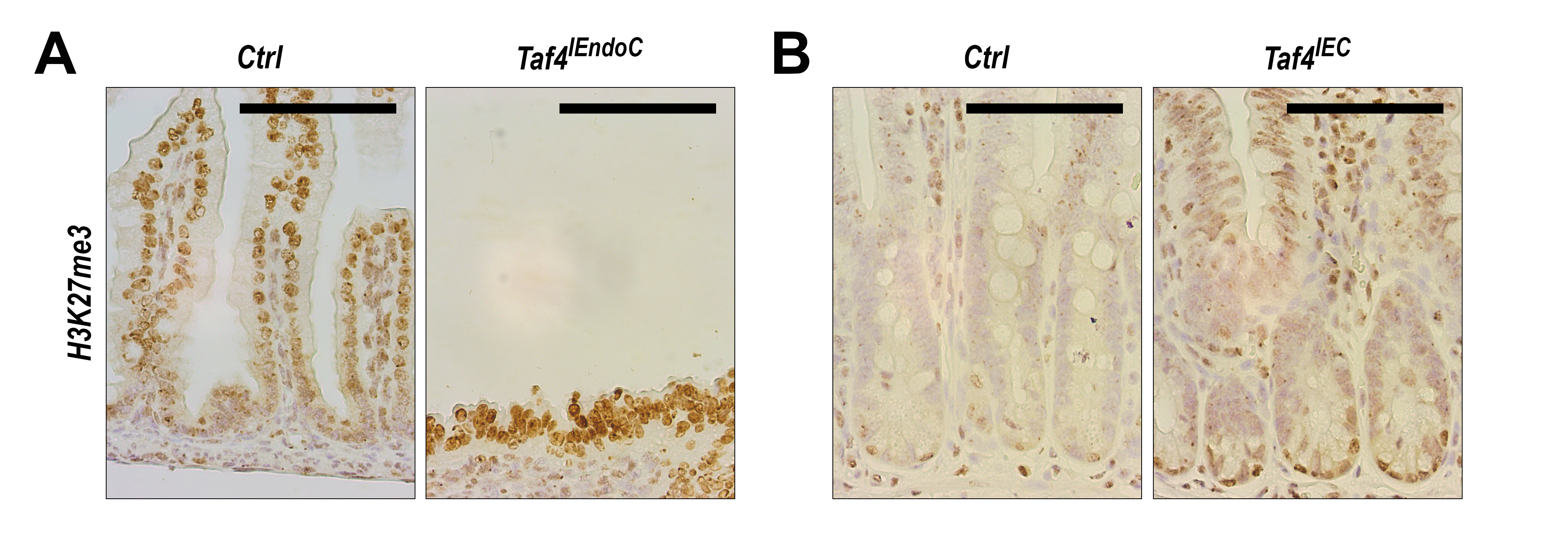

Supplement: Supplementary file 7 — Supp Fig 6 [file 41418_2022_1109_MOESM7_ESM.tif]
